# Beyond Catalytic Therapy: Copper‐Paeonol Nanozymes Disrupt Fascin‐Mediated Actin Bundling to Suppress Tumor Growth and Metastasis

**DOI:** 10.1002/advs.202512186

**Published:** 2025-12-17

**Authors:** Peiying Zhang, Huajun Li, Yisen Wang, Jie Xiang, Lei Fan, Shengzhe Zhang, Lizeng Gao, Hua Dai, Juqun Xi

**Affiliations:** ^1^ School of Traditional Chinese Medicine Faculty of Medicine Yangzhou University Yangzhou Jiangsu 225009 China; ^2^ Key Laboratory of the Jiangsu Higher Education Institutions for Integrated Traditional Chinese and Western Medicine in Senile Diseases Control (Yangzhou University) Yangzhou Jiangsu 225009 China; ^3^ School of Chemistry and Materials Yangzhou University Yangzhou Jiangsu 225002 China; ^4^ CAS Engineering Laboratory for Nanozyme Key Laboratory of Biomacromolecules Institute of Biophysics Chinese Academy of Sciences Beijing 100101 China

**Keywords:** actin‐bundling activity, catalytic therapy, fascin, metal‐phenolic nanozymes, tumor metastasis

## Abstract

Fascin, an actin‐bundling protein universally upregulated in metastatic tumors, drives tumor migration and invasion by promoting filopodia and invadopodia formation, establishing it as a pivotal therapeutic target. Herein, copper‐paeonol nanozymes (CuPaeNs) is engineered through metal‐phenolic complexation, mimicking natural enzyme metal‐coordination microenvironments to confer peroxidase‐like activity. This enzymatic capability drives the conversion of tumor‐associated H_2_O_2_ into cytotoxic hydroxyl radicals, inducing oxidative damage in malignant cells. Notably, beyond inducing tumor catalytic therapy via targeted ROS generation, CuPaeNs directly disrupted the actin‐bundling activity of fascin, as evidenced by molecular docking, isothermal titration calorimetry, co‐immunoprecipitation, and immunofluorescence assays. Transcriptomic and biochemical analyses further revealed that CuPaeNs suppressed melanoma glycolysis by blocking the fascin‐YAP1‐PFKFB3 signaling axis. This study establishes metal‐phenolic nanozymes as a dual‐functional strategy that simultaneously triggers ROS overproduction to amplify tumor oxidative stress and disrupts fascin‐mediated metastasis, thereby modulating tumor metabolic reprogramming. This coordinated intervention establishes a novel treatment framework for malignancies characterized by fascin overexpression.

## Graphical abstract

Copper‐paeonol nanozymes target tumor‐specific reactive oxygen species generation and disrupt fascin‐mediated actin bundling, effectively suppressing tumor growth and metastatic colonization.

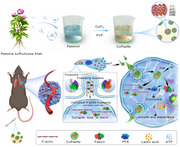

## 1 Introduction

Metastasis, the spread of tumor cells to distant organs from their site of origin, represents the most advanced and deadliest stage of a tumor.^[^ ^1^ ^]^ Unlike primary tumors, which may be effectively treated with localized surgery or radiation, metastasis is a systemic disease.^[^ ^2^ ^]^ Once tumors metastasize, they possess the capability to rapidly become life‐threatening.^[^ ^3^, ^4^ ^]^ Therefore, the imperative for ongoing research into tumor metastasis is increasingly pressing and crucial.^[^ ^5^ ^]^ However, comprehending the mechanisms underlying tumor metastasis and developing effective strategies to combat it remain significant challenges.^[^ ^6^, ^7^ ^]^ The migration and invasion of tumor cells are crucial stages in metastasis. In order for metastasis to occur, actin within tumor cells must reorganize the cytoskeleton by forming actin bundles, thereby altering cell shape.^[^ ^8^, ^9^ ^]^ Actin filaments themselves are flexible and incapable of generating membrane protrusions. However, bundles of actin filaments provide rigidity, resisting pressure from the plasma membrane.^[^ ^10^, ^11^ ^]^ Fascin, the filamentous actin (F‐actin) binding protein, is a pivotal molecule involved in cell motility.^[^ ^12^ ^]^ It facilitates the cross‐linking of individual actin filaments into straight, dense, and rigid bundles, which are essential for the formation of stereocilia, filopodia, and other finger‐like membrane protrusions, thereby playing a critical role in promoting metastatic progression.^[^ ^13^ ^]^ Briefly, there are three isoforms of fascin (fascin‐1, ‐2, and ‐3) in animals,^[^ ^14^, ^15^ ^]^ with high levels of fascin‐1 (FSCN1) significantly associated with tumor invasiveness.^[^ ^12^, ^13^, ^14^, ^16^ ^]^ Besides its well‐established role in metastatic dissemination, fascin also boosts resistance against metabolic stress, tumor cell stemness, and chemotherapy.^[^ ^17^, ^18^ ^]^ Therefore, fascin is recognized as a promising therapeutic target for inhibiting tumor metastasis. For example, NP‐G2‐044, a fascin inhibitor developed by Novita Pharmaceuticals, Inc., is currently in Phase II clinical trials. It can target and bind to fascin, thereby preventing the interaction between fascin and actin filaments.^[^ ^11^ ^]^ The prevention of actin bundling and filopodia formation exhibits significant anti‐tumor efficacy.^[^ ^19^ ^]^ However, as an orally active drug, NP‐G2‐044 acts quickly but has a short half‐life, requiring multiple daily oral administrations, leading to poor patient compliance. Thus, more research is needed to find new ways to target fascin with greater efficacy.

Metal‐phenolic nanozymes are synthesized through the coordinated self‐assembly of phenolic ligands with metal ions (e.g., Fe^3+^, Cu^2+^ or Mn^2+^), exploiting their intrinsic metal‐phenolic coordination chemistry.^[^ ^20^, ^21^ ^]^ This bottom‐up approach allows precise modulation of nanozyme's morphology and catalytic activity by adjusting synthesis parameters such as pH, metal‐to‐ligand ratios, and templating agents. By structurally mimicking the active sites of natural metalloenzymes, these nanozymes demonstrate programmable enzyme‐like activities, including peroxidase (POD), oxidase (OXD), and catalase (CAT) functionalities,^[^ ^22^, ^23^ ^]^ enabling precise regulation reactive oxygen species (ROS) in biological environments.^[^ ^24^ ^]^ Paeonol (Pae), a bioactive phenolic compound derived from *Paeonia suffruticosa* root bark,^[^ ^25^ ^]^ has demonstrated broad‐spectrum anticancer effects in preclinical models by inhibiting tumor cell proliferation, migration, and invasion.^[^ ^26^, ^27^ ^]^ However, its clinical utility is hampered by inherent limitations such as rapid metabolism, poor aqueous solubility, and low bioavailability, often requiring high therapeutic doses that exacerbate systemic toxicity.^[^ ^28^ ^]^ To overcome these challenges, researchers are leveraging metal‐phenolic coordination strategies. The phenolic hydroxyl group in paeonol enable chelation with transition metals, forming stable metal‐paeonol complexes that enhance its physicochemical stability and bioactivity.^[^ ^29^, ^30^ ^]^ Copper, a physiologically essential trace metal, plays dual roles in biological systems. It serves as a cofactor for redox‐active enzymes (e.g., cytochrome c oxidase) and modulates cell signaling pathways through copper‐binding proteins.^[^ ^31^ ^]^ Recent advances have uncovered novel copper‐dependent therapeutic mechanisms, including the copper‐induced cell death pathway termed “cuproptosis”, which is triggered by proteotoxic stress from copper overload.^[^ ^32^ ^]^ Therefore, by integrating the catalytic prowess of copper‐paeonol nanozymes with the intrinsic bioactivity of both the copper ion and the organic ligand, this hybrid system offers a unique platform for uncovering novel therapeutic mechanisms.

**Figure 1:**
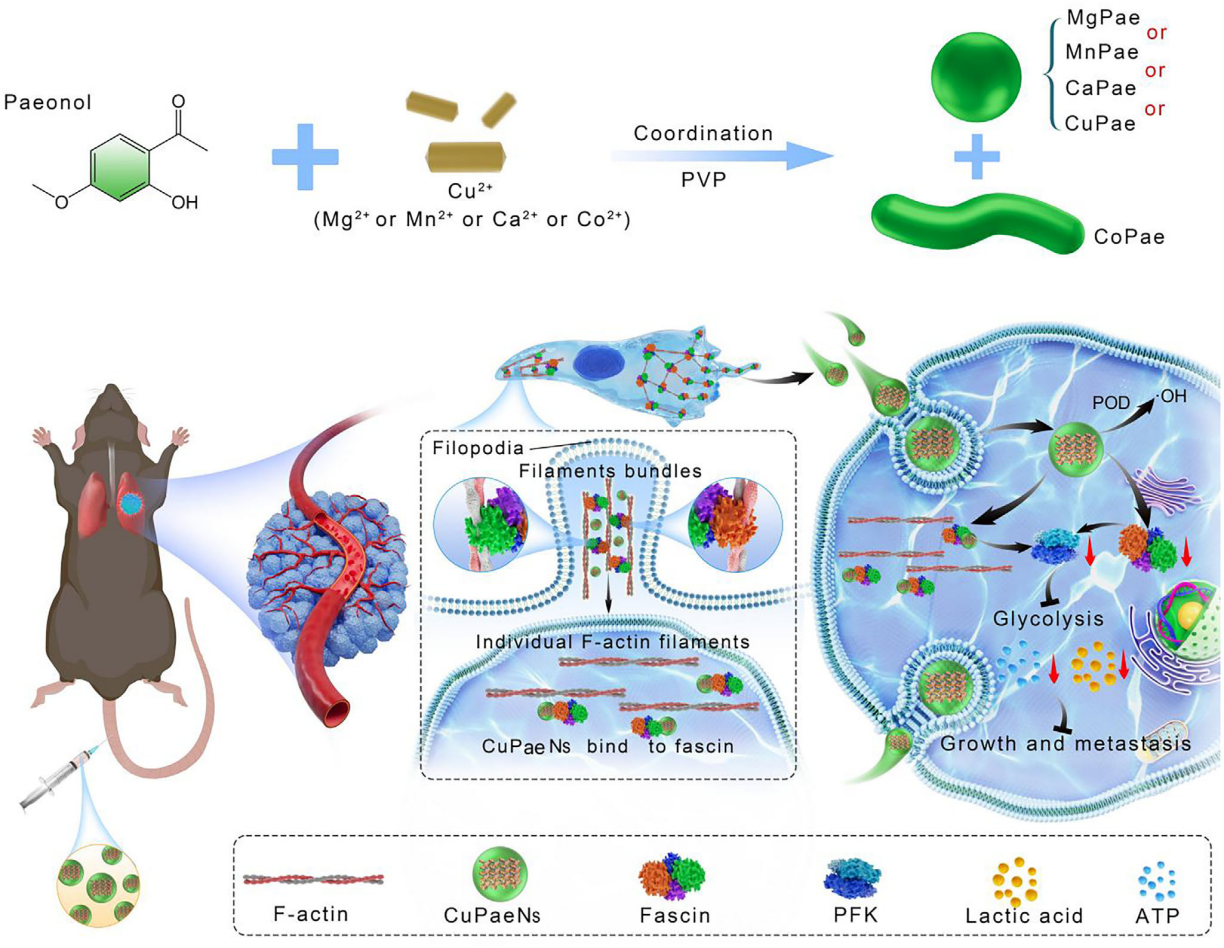
Schematic illustration. A) Scheme for the self‐assembly of Cu^2+^, Mg^2+^, Mn^2+^, Ca^2+^ or Co^2+^ with paeonol to form different nanostructures. B) CuPaeNs triggering oxidative damage and disrupting fascin‐mediated actin bundling for tumor therapy.

In this study, we developed copper‐paeonol nanozymes (CuPaeNs) by biomimetically replicating the metal‐coordination microenvironment of metalloenzymes through metal‐phenolic ligand assembly. The resulting CuPaeNs exhibited robust POD‐like activity, enabling tumor‐specific catalytic therapy via conversion of tumor‐associated H_2_O_2_ into cytotoxic •OH. Notably, CuPaeNs induced a morphological shift in tumor cells from spindle‐shaped to rounded, phenocopying the effects of the fascin‐specific inhibitor NP‐G2‐044. Importantly, among a series of metal‐paeonol assemblies (MnPae, MgPae, CaPae, CoPae, and CuPae), only CuPaeNs demonstrated unique targeting capability toward fascin‐dependent actin‐bundling activity. Mechanistically, this phenotype correlated with downregulation of fascin expression and disruption of fascin‐actin filament interactions, suggesting dual chemo‐catalytic and cytoskeletal targeting capabilities of CuPaeNs. To validate these findings, we selected highly metastatic melanoma as a model system. In vitro and in vivo experiments demonstrated that CuPaeNs coordinately induced the oxidative damage and disrupted the actin‐bundling activity of fascin, effectively suppressing melanoma cell growth and metastasis. Further mechanistic studies revealed that CuPaeNs inhibited glycolysis in melanoma cells by modulating the fascin‐YAP1‐PFKFB3 signaling axis, thereby blocking energy metabolism essential for metastatic progression (**Figure** **1**). Importantly, the therapeutic efficacy of CuPaeNs extended beyond melanoma, as evidenced in breast mouse models. Collectively, this work establishes a new metal‐phenolic nanozyme platform that synergizes catalytic ROS generation with fascin‐targeted cytoskeletal remodeling, offering a translational rationale for the development of fascin‐directed nanozyme therapeutics against tumors overexpressing fascin.

## 2 Results

### 2.1 Self‐Assembly of Cu^2+^‐Chelating Paeonol Nanozymes

**Figure 2:**
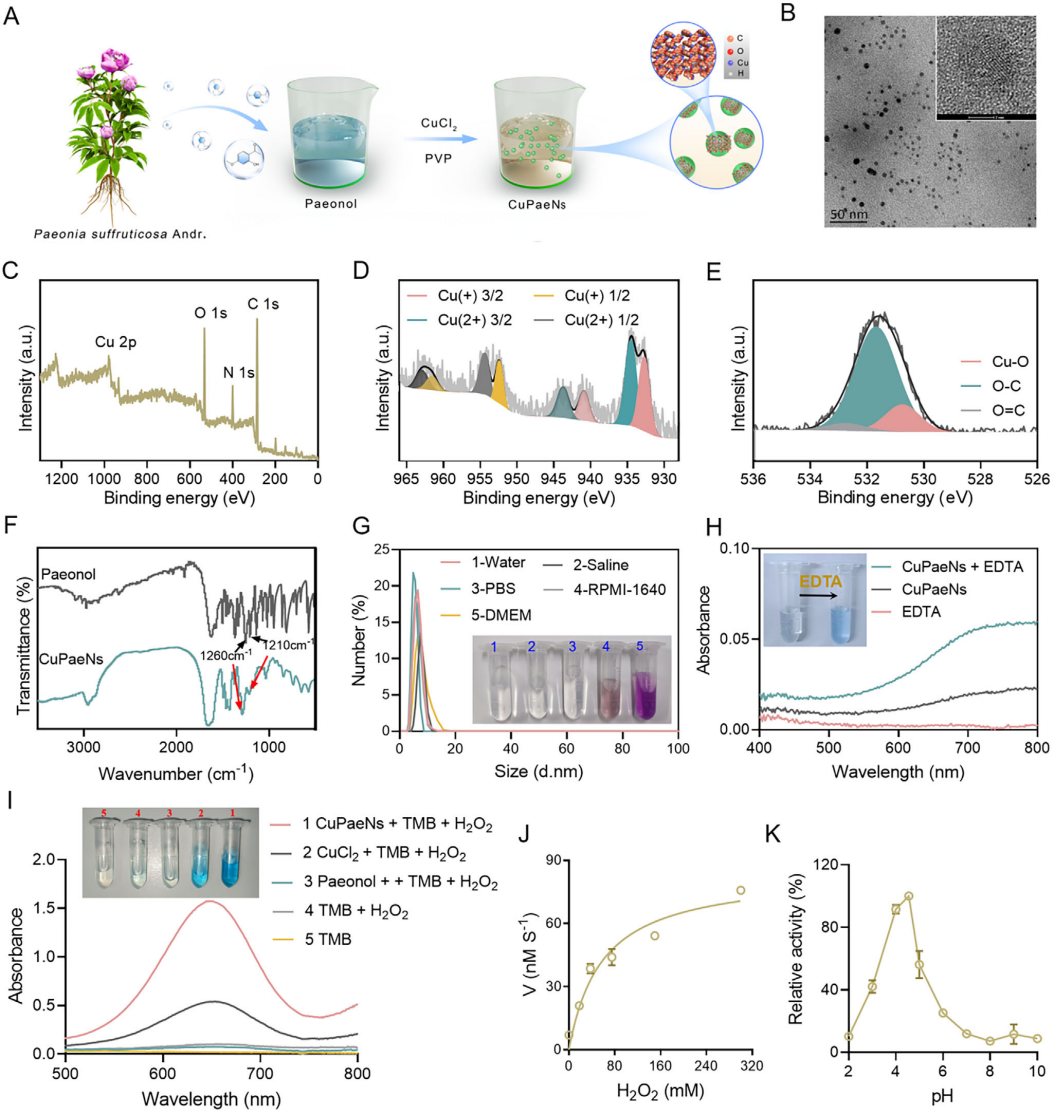
Structural characteristics and POD‐like activity of CuPaeNs. A) Schematic illustration of CuPaeNs synthesis. B) TEM image of CuPaeNs. C) XPS patterns of CuPaeNs. D,E) High‐resolution elemental XPS spectra for Cu D) and O E). F) FTIR spectra of paeonol and CuPaeNs. G) DLS results of CuPaeNs in different media. H) UV–Vis spectra of CuPaeNs before and after EDTA treatment (inset: digital photographs of color changes). I) UV–Vis absorbance spectra and color changes of TMB in different reaction systems. J) Kinetic assay for the catalytic activity of CuPaeNs with H_2_O_2_ as the substrate (*n* = 3). K) pH‐dependent catalytic activity of CuPaeNs (*n* = 3).

Copper‐paeonol nanozymes (CuPaeNs) were prepared following the procedure outlined in **Figure** **2**A. Following dialysis and freeze‐drying of the mixture, CuPaeNs exhibiting a chartreuse coloration were obtained (Figure S1, Supporting Information). This notable change in color from blue (CuCl_2_) to chartreuse (CuPaeNs) indicated the occurrence of interaction between Cu^2+^ and paeonol. Transmission electron microscopy (TEM) image (Figure 2B) revealed that the CuPaeNs were uniformly dispersed with an average diameter of 4 nm, while the X‐ray diffraction (XRD) analysis confirmed their amorphous nature (Figure S2, Supporting Information). Additional crucial details from Figure 1C–E were obtained through X‐ray photoelectron spectroscopy (XPS) analysis, which characterized the surface chemical composition and elemental states of CuPaeNs. The analysis confirmed the presence of C, N, O, and Cu in the CuPaeNs at atomic percentages of 62.8%, 11.9%, 15.8%, and 1.8%, respectively (Figure 2C). The predominant ionic state of copper in CuPaeNs was Cu^2+^, as indicated by the Cu*2p* XPS spectrum, with a partial amount of Cu^+^ reduced by paeonol also present (Cu^2+^:Cu^+^ = 1.2:1) (Figure 2D). Specifically, confirmation of Cu chelation was further supported by analysis of the O*1s* spectrum, revealing a Cu–O peak observed at 530.7 eV (Figure 2E).^[^ ^33^ ^]^ Analysis of the Fourier transform infrared (FTIR) spectroscopy in Figure 2F indicated a change in the characteristic peak at 1210–1260 cm^−1^ (HO─C stretching band) and a reduction in infrared intensity, also suggesting the coordination between copper ions and the HO–C groups of paeonol.^[^ ^34^ ^]^ Subsequently, the hydrodynamic diameters of CuPaeNs dispersed in various media, such as water, saline, phosphate‐buffered saline (PBS), cell culture media (Dulbecco's Modified Eagle Medium (DMEM), and Roswell Park Memorial Institute‐1640 (RPMI‐1640)), were assessed using dynamic light scattering (DLS) measurements (Figure 2G). For instance, in PBS, CuPaeNs exhibited an average hydrodynamic diameter of 6.5 nm, with zeta potentials of −6.7 mV. Importantly, CuPaeNs exhibited excellent stability, showing no significant changes in hydrodynamic diameter under weakly acidic conditions (pH 2.0–7.0) and after one week of long‐term storage (Figure S3, Supporting Information). Moreover, the addition of ethylenediaminetetraacetic acid (EDTA) induced a distinct color change in the CuPaeNs solution from colorless to vibrant blue (inset, Figure 2H), accompanied by significant alterations in the UV–Vis absorption spectra (Figure 2H). This spectral shift reflects the disassembly of copper ions from CuPaeNs, resulting from their coordination with EDTA to form more stable metal‐EDTA complexes. Together, these findings demonstrate that copper ions are coordinated to paeonol in CuPaeNs, which underpins their high stability under physiological conditions.

By structurally mimicking the catalytic centers of natural metalloenzymes, the engineered CuPaeNs exhibited significant enzyme‐mimetic activity. As shown in Figure 2I, CuPaeNs could induced a color reaction of the substrate 3, 3, 5, 5‐tetramethylbenzidine (TMB) in the presence of H_2_O_2_, demonstrating POD‐mimetic activity. Notably, at an equivalent copper concentration, the metal‐paeonol coordination architecture endowed CuPaeNs with enhanced POD‐like activity, outperforming free Cu^2+^ ions by ≈3.0‐fold in TMB oxidation absorbance. Then, using H_2_O_2_ as the substrate, the key enzyme kinetic parameters of CuPaeNs, Michaelis constant (*K*_m_) and maximal reaction velocity (*V*_max_), were calculated as 58.3 mm and 84.0 nm s^−1^, respectively (Figure 2J). The *K*_m_ and *V*_max_ values of CuPaeNs are comparable to those of other copper‐containing nanozymes (Table S1, Supporting Information), indicating its efficacy as a POD mimic capable of modulating redox homeostasis in biological systems through intrinsic enzyme‐like activity. Meanwhile, the catalytic activity of CuPaeNs, like other POD mimics, was dependent on pH and temperature, reaching optimal efficiency at pH 4.5 and 37 °C (Figures 2K; S4, Supporting Information). Such pH/temperature‐responsive characteristics allow CuPaeNs to effectively exert their POD‐like activity upon reaching the acidic tumor microenvironment (TME). Furthermore, the electron spin resonance (ESR) spectroscopy directly verified hydroxyl radical (•OH) generation by CuPaeNs, confirming their catalytic activity (Figure S5, Supporting Information). Taken together, CuPaeNs synthesized via metal‐ligand coordination‐driven self‐assembly exhibit intrinsic POD‐mimetic activity, enabling tumor‐specific catalytic therapy through targeted ROS generation.

### 2.2 CuPaeNs Exhibited Catalytic ROS‐Dependent Cytotoxicity In Vitro

**Figure 3:**
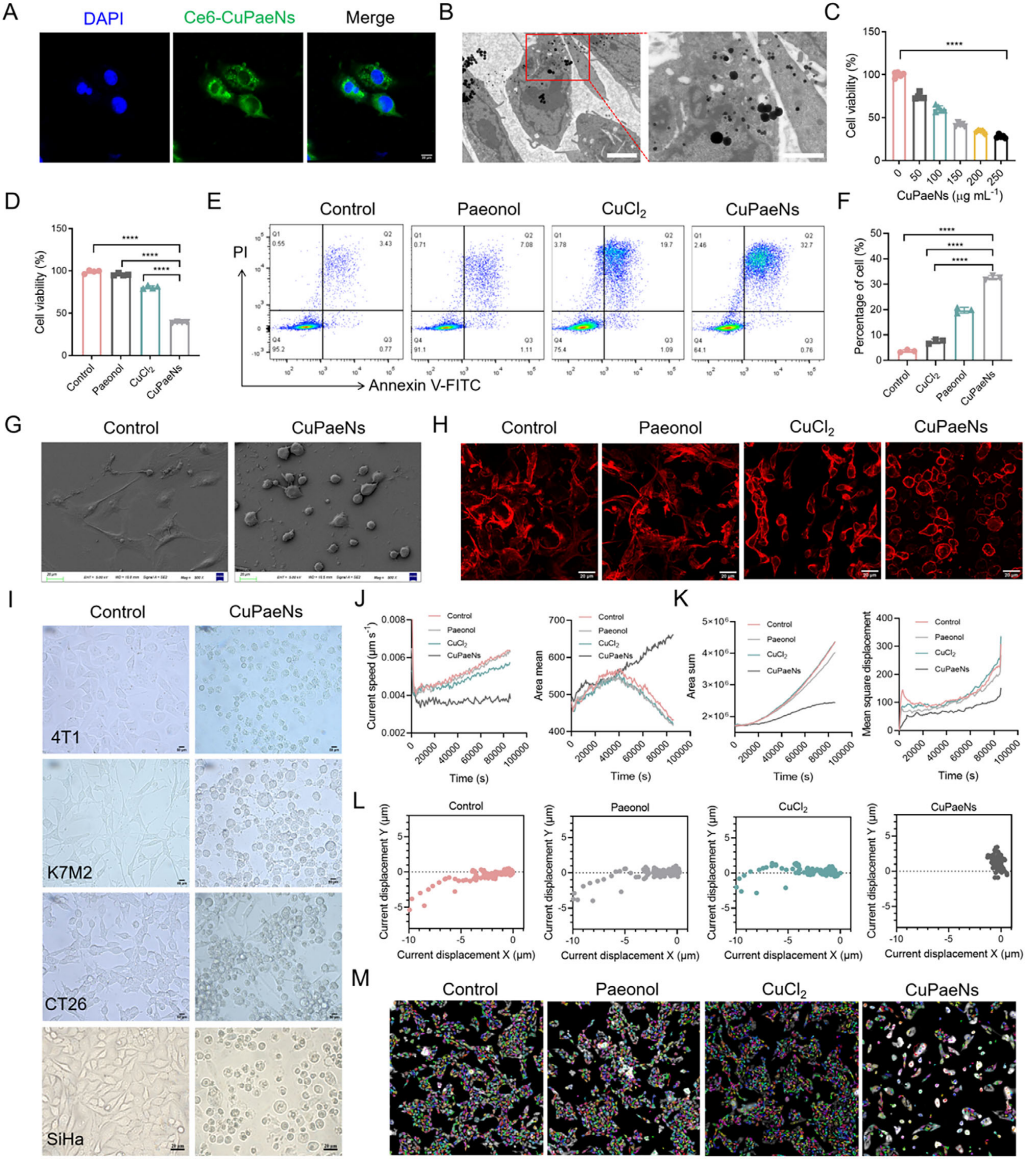
CuPaeNs inhibited the growth and induced cytomorphological alterations of tumor cells. A) CLSM images of B16 cells after incubation with Ce6‐CuPaeNs, scale bar = 20 µm. B) Intracellular localization of CuPaeNs in B16 cells, observed by TEM, scale bar = 2.0 and 1.0 µm, respectively. C) Viability of B16 cells treated with various concentrations of CuPaeNs (*n* = 5). D) Comparison of the cytotoxic effects of paeonol, CuCl_2_, and CuPaeNs on B16 cells (*n* = 4). Paeonol and CuCl_2_ were used at equivalent concentrations to CuPaeNs. E) Scatter plot demonstrating B16 cell apoptosis and F) corresponding quantitative analysis following different treatments using flow cytometry (*n* = 3). G) SEM images of B16 before and after the CuPaeNs treatment, scale bar = 20 µm. H) CLSM images of F‐actin with B16 cells after different treatments, scale bar = 10 µm. I) Bright‐field microscopy analyses of different tumor cells before and after the CuPaeNs treatment, scale bar = 50 µm. J) Using well‐level data, the current speed and mean square displacement in different groups were plotted against observation time. K) Area sum and area mean in different groups were plotted against observation time. L) Visualization of cell displacement in different groups. M) Real‐time dynamic displacement of B16 cells in different groups observed at 24 h. Statistically significant differences are indicated with their respective *p*‐values (^****^ *p* <0.0001).

Given the pH‐dependent POD‐like activity of CuPaeNs, we investigated their subcellular localization in tumor cells, where distinct organelles exhibit varying pH microenvironments. Initially, cellular uptake of CuPaeNs in B16 murine melanoma cells was analyzed via confocal laser scanning microscopy (CLSM) and TEM. To enable tracking, CuPaeNs were conjugated with chlorin e6 (Ce6) to generate fluorescent Ce6‐CuPaeNs. CLSM imaging revealed the intracellular accumulation of Ce6‐derived green fluorescence (**Figure** **3**A), while TEM results confirmed the lysosomal localization of CuPaeNs (Figure 3B). Furthermore, the lysosomal escape behavior of CuPaeNs was evaluated using a co‐localization assay. Briefly, B16 cells were co‐stained with LysoTracker Green (lysosomes) and Rhodamine‐labeled CuPaeNs, and then imaged by CLSM at 3, 6, and 9 h post‐incubation. The co‐localization extent was quantified using Pearson's correlation coefficient (PCC), with higher values indicating stronger co‐localization. As shown in Figure S6 (Supporting Information), the PCC between CuPaeNs and LysoTracker Green decreased significantly over time dropping to 0.29 at 9 h post‐incubation, indicating efficient lysosomal escape. Within the acidic lysosomal compartment (pH ≈5.0), the POD‐like activity of CuPaeNs is optimally activated, promoting ROS accumulation and leading to cellular damage. After escaping into the cytoplasm, CuPaeNs have the potential to exert additional biological functions. Consistent with this mechanism, the levels of ROS in CuPaeNs‐treated B16 cells were initially measured using the fluorescent probe 2′, 7′‐dichlorodihydrofluorescein diacetate (DCFH‐DA). As shown in Figure S7 (Supporting Information), CuPaeNs‐treated cells exhibited a marked increase in intracellular green fluorescence intensity relative to untreated controls. Notably, neither free paeonol nor CuCl_2_ at equivalent concentrations induced significant oxidative stress alterations. To further evaluate the therapeutic implications, we systematically analyzed the cytotoxicity profile of CuPaeNs. Dose‐response assays demonstrated a clear cytotoxic dependency of CuPaeNs (Figure 3C). Strikingly, CuPaeNs exhibited substantially enhanced cytotoxicity compared to both free paeonol and CuCl_2_ (Figure 3D), aligning with its ROS‐elevating capacity. The pro‐apoptotic activity of CuPaeNs was quantitatively assessed using Annexin V‐FITC/PI dual staining coupled with flow cytometry. As shown in Figure 3E,F, while paeonol and CuCl_2_ individually induced moderate apoptosis, CuPaeNs demonstrated a synergistic effect, significantly amplifying apoptotic rates compared to either component alone. Furthermore, Hoechst/Propidium Iodide (PI) co‐staining corroborated these findings evidenced by increased PI‐positive permeabilized cells in CuPaeNs‐treated group (Figure S8, Supporting Information). In addition, the mitochondrial membrane potential (MMP) of B16 cells before and after CuPaeNs treatment was assessed using 5, 5′, 6, 6′‐tetrachloro‐1, 1′, 3, 3′‐tetraethylimidacarbocyanine (JC‐1) staining. As illustrated in Figure S9 (Supporting Information), a marked depolarization of MMP, a hallmark event initiating intrinsic apoptosis,^[^ ^35^ ^]^ was observed in CuPaeNs‐treated B16 cells compared to untreated controls. Furthermore, upon incubation with B16 cells, the CuPaeNs depleted the local glutathione (GSH) (Figure S10, Supporting Information), a key antioxidant for cancer cell survival.^[^ ^36^ ^]^ XPS analysis confirms the coexistence of Cu⁺ and Cu^2^⁺ in CuPaeNs (Figure 2D). As previously reported,^[^ ^37^ ^]^ Cu⁺ species contribute efficiently to •OH generation through its high catalytic activity, while Cu^2^⁺ depletes GSH by being reduced to Cu⁺. This mechanism enables CuPaeNs to effectively enhance ROS accumulation in the tumor microenvironment, creating synergistic oxidative stress essential for catalytic tumor therapy. Importantly, the broad‐spectrum cytotoxicity of CuPaeNs was further validated across multiple cancer cell types, including mammary adenocarcinoma (4T1), murine osteosarcoma (K7M2), colorectal carcinoma (CT26), and human cervical carcinoma (SiHa), demonstrating consistent antiproliferative effects independent of tissue origin (Figure S11, Supporting Information). Taken together, these results establish that CuPaeNs act as a POD‐mimetic nanozyme that utilizes endogenous H_2_O_2_ in the tumor microenvironment to drive potent antitumor effects via catalytic therapy.

### 2.3 CuPaeNs Triggered Cytomorphological Alterations in Tumor Cells

Notably, during cytotoxicity assessment of CuPaeNs, we observed distinct morphological alterations in tumor cells post‐treatment. As shown in Figures 3G and S12 (Supporting Information), scanning electron microscopy (SEM) and bright‐field microscopy analyses revealed a cytomorphological transition from the characteristic spindle‐like morphology of untreated cells to a rounded, contracted phenotype in CuPaeNs‐treated B16 cells. This structural remodeling, potentially indicative of cytoskeletal collapse, was not observed in CuCl_2_‐ or paeonol‐treated groups, highlighting the specificity of CuPaeNs‐induced cytotoxicity. To clearly visualize cytoskeletal collapse, we performed phalloidin staining of filamentous actin (F‐actin). As shown in Figure 3H, CuPaeNs‐treated cells exhibited a marked reduction in filopodia and lamellipodia formation, indicating cytoskeletal destabilization preceding apoptosis. In contrast, treatment with paeonol or CuCl_2_ induced no significant alterations in cytoskeletal architecture, which aligned with their reduced cytotoxic effects. Notably, similar morphological alterations were consistently observed across multiple cancer cell lines (4T1, K7M2, CT26, and SiHa) (Figure 3I), corroborating the broad‐spectrum cytoskeletal destabilization induced by CuPaeNs. To determine the specificity of the observed phenomenon to CuPaeNs, we synthesized comparative metal‐paeonol assemblies, including MnPae (Mn^2+^‐paeonol), CoPae (Co^2+^‐paeonol), MgPae (Mg^2+^‐paeonol), and CaPae (Ca^2+^‐paeonol). As illustrated in Figure S13 (Supporting Information), although paeonol coordinated with diverse metal ions to form nanostructures, substantial variations existed in their morphological characteristics and dimensional parameters. Notably, MnPae, MgPae, and CaPae exhibited minimal anti‐tumor efficacy and failed to induce morphological changes in B16 cells, as evidenced by Figure S14 (Supporting Information). Although CoPae exhibited inherent cytotoxicity, it did not induce the distinct cytoskeletal reorganization characteristic of CuPaeNs treatment (Figure S15, Supporting Information). These findings demonstrate that CuPaeNs exhibit tumor cell‐specific targeting capability, and the distinctive effect of CuPaeNs on cytomorphological alterations drives further mechanistic exploration of cytoskeletal architectures.

Building upon the well‐characterized mechanistic link between cytoskeletal remodeling and malignant progression, we systematically investigated the anti‐metastatic effects of CuPaeNs. First, a wound‐healing migration assay revealed that CuPaeNs (3.2% healing ratio) significantly inhibited B16 cells migration compared to paeonol (22.2%), CuCl_2_ (30.0%), and PBS controls (33.2%) after 24 h (Figure S16, Supporting Information). Similarly, transwell invasion assays demonstrated a marked reduction in invaded B16 cells following CuPaeNs treatment (Figure S17, Supporting Information). High‐content imaging analysis (PerkinElmer Operetta CLS) tracked real‐time cell motility over 24 h (Figure 3J,K). CuPaeNs‐treated cells exhibited progressive decreases in instantaneous velocity and mean square displacement (quantified displacement magnitude averaged across all cells) compared to controls. Concurrently, total cell area measurements revealed minimal surface coverage in the CuPaeNs group, consistent with growth suppression. Cell displacement trajectories (Figure 3L) and 24 h dynamic tracking (Figure 3M and Video S1, Supporting Information) confirmed CuPaeNs’ superior efficacy in blocking B16 cells migration relative to paeonol and CuCl_2_. Collectively, these data demonstrate that CuPaeNs not only suppress B16 cells proliferation but also potently inhibit metastatic behaviors, including migration and invasion, likely mediated by their effects on actin cytoskeletal remodeling.

### 2.4 CuPaeNs Modulated FSCN1‐Mediated Actin Bundling Activity

**Figure 4:**
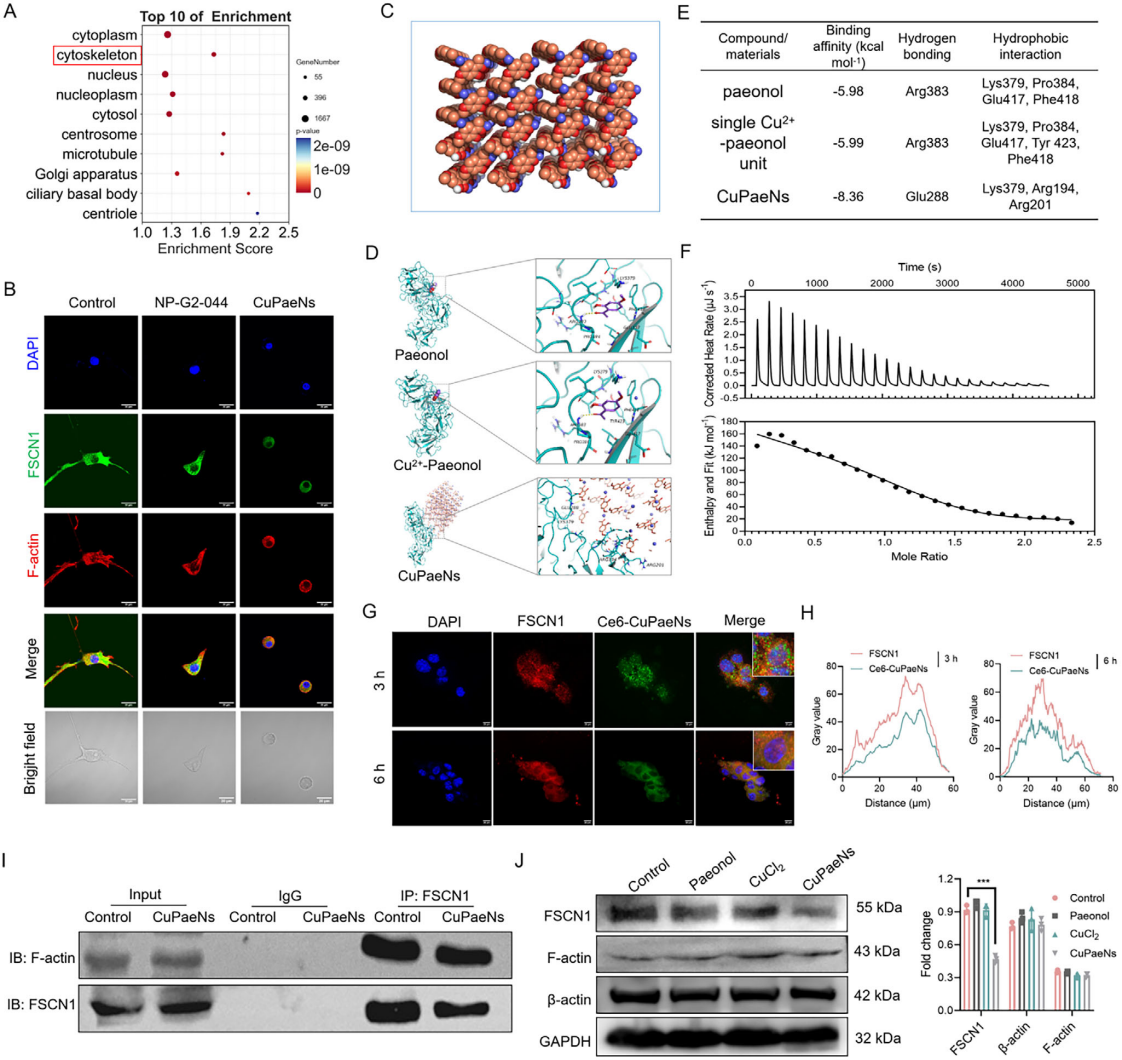
CuPaeNs bound to FSCN1. A) Bubble chart of GO enrichment analysis top 10 of cellular component after CuPaeNs treatment. B) Immunofluorescence staining of B16 cells in different groups. DAPI (blue), FSCN1 (green), and F‐actin (red). C) Molecular simulation of CuPaeNs. D) Computational model of active sites related to the potential key target of FSCN1 with paeonol, a single paeonol‐Cu^2+^ unit, and CuPaeNs. The residues of the ligand‐binding domain proteins, involved in hydrogen bonds, were illustrated and marked with yellow dotted lines. E) Molecular simulation illustrating the binding affinity and detailed intermolecular binding interactions of FSCN1 with paeonol, a single Cu^2+^‐paeonol unit, and CuPaeNs, focusing on main hydrogen bonds and hydrophobic interactions. F) Isothermal titration calorimetry result demonstrating the direct binding of CuPaeNs to FSCN1. G) CLSM images showing intracellular FSCN1 binding with CuPaeNs in B16 cells (scale bar = 20 µm) and H) responding pixel intensity plot. I) Assessment of CuPaeNs's impact on FSCN1's actin‐bundling activity using the Co‐IP assay. (J) Protein expression of FSCN1, F‐actin, and β‐actin with B16 cells in different groups, along with quantitative analysis (*n* = 3). Statistically significant differences are indicated with their respective *p*‐values (^***^ *p* < 0.001).

By integrating analyses of morphological alterations, cytotoxic responses, and anti‐metastatic outcomes, we propose that CuPaeNs exert their antitumor effects not only through amplification of TME oxidative stress, but also via coordinated targeting of cytoskeletal proteins to simultaneously block cellular proliferation and metastatic dissemination. The RNA sequencing (RNA‐seq)‐based Gene Ontology enrichment analysis, conducted with dual selection parameters (expression threshold: PopHits ≥ 5; significance threshold: ‐log10[p‐value] > 3.0), identified cytoskeleton‐related genes as the most significantly enriched group within the cellular component ontology upon CuPaeNs exposure (**Figure** **4**A). Given fascin's established role as a key cytoskeletal regulator of metastatic cell migration and invasion, we performed mechanistic interrogation of CuPaeNs‐fascin interaction through structural and functional analyses to elucidate its targeting potential.

We first conducted a comparative study of CuPaeNs against NP‐G2‐044, a clinical‐stage fascin inhibitor exhibiting validated suppression of F‐actin bundling activity.^[^ ^11^ ^]^ As shown in Figure 4B, triple immunofluorescence staining was performed using blue‐fluorescent DAPI (4′, 6‐diamidino‐2‐phenylindole) for nuclei, green‐fluorescent anti‐FSCN1 for fascin, and red‐fluorescent phalloidin for F‐actin architecture visualization. The CLSM analysis of filopodial dynamics revealed striking morphological differences. Untreated B16 control cells exhibited abundant filopodia, characteristic of metastatic motility. NP‐G2‐044 treatment induced significant cytoskeletal remodeling, reducing filopodia density and shortening protrusion length. Crucially, CuPaeNs demonstrated comparable filopodia suppression, confirming shared fascin‐mediated antimetastatic mechanisms with the clinical inhibitor. In addition, CuPaeNs demonstrated superior efficacy in inducing filopodia formation relative to individual paeonol or CuCl_2_ treatments (Figure S18, Supporting Information). Similar CLSM findings in 4T1 cells were shown in Figure S19 (Supporting Information). Then, to figure out the function of CuPaeNs to FSCN1, we performed molecular docking of CuPaeNs with FSCN1 to assess its ability to disrupt the actin‐bundling activity of FSCN1. Given the negligible impact of PVP stabilizers on cellular architecture (Figure S20, Supporting Information), we conclusively assigned the core structure to Cu^2+^ ions chelated by paeonol through phenolic hydroxyl groups. As illustrated in Figure 4C, each copper ion can bind to one paeonol molecule, resulting in a complex with a size of ≈4 nm. For comparison, paeonol and a single paeonol‐Cu^2+^ unit were also included in the docking analysis with bundling. Molecular simulations were conducted to predict the type of interaction force and binding affinity for paeonol, a single paeonol‐Cu^2+^ unit, and CuPaeNs binding to FSCN1. From Figure 4D,E, it was evident that all compounds demonstrated strong binding properties to FSCN1 with an affinity of less than −5.0 kcal mol^−1^. Crucially, our findings revealed that CuPaeNs demonstrated a stronger binding affinity to FSCN1 compared to paeonol and the single paeonol‐Cu^2+^ unit. Specifically, paeonol and the single paeonol‐Cu^2+^ unit both formed hydrogen bonds with FSCN1 at the Arg383 residue, and paeonol‐FSCN1 and single paeonol‐Cu^2+^ unit‐FSCN1 had four and five hydrophobic contacts, respectively, involving Lys379, Pro384, Glu417, Phe418, and Lys379, Pro384, Glu417, Tyr 423, Phe418. Meanwhile, CuPaeNs was found to form hydrogen bonds with FSCN1 at the Glu288 residue, and the interaction between CuPaeNs and FSCN1 involved three hydrophobic contacts at Lys379, Arg194, and Arg201. The N‐terminal and C‐terminal residues of FSCN1 encompass two adjacent actin‐binding surfaces that interact with a single F‐actin: one between β‐trefoil 1 and β‐trefoil 4 (actin‐binding surface 1, ABS1), and the other between β‐trefoil 1 and β‐trefoil 2 (ABS2).^[^ ^38^ ^]^ Residues Arg383 and Glu288, located near the actin‐binding domain ABS1, are involved in actin aggregation and are considered ideal targets for tumor therapy. By simulation calculation, we concluded that all compounds formed hydrophobic interactions on Lys379, which proved to be the key residue with strong actin clustering activity at the actin‐binding site 3.^[^ ^39^ ^]^ Together, the synthesized CuPaeNs demonstrated potent inhibition of FSCN1 by forming hydrogen bonds with the actin‐binding region ABS1 and the key active site Glu288.

Subsequently, we employed isothermal calorimetry (ITC) to ascertain the direct interaction between FSCN1 and CuPaeNs. ITC is a technique used to measure the heat released or absorbed during molecular interactions. It is employed to determine the thermodynamic parameters of interactions between molecules, typically small molecules and proteins.^[^ ^11^, ^40^ ^]^ As depicted in Figure 4F, the raw data consisted of a series of heat flow peaks, with each peak corresponding to a single injection of CuPaeNs into the FSCN1 solution. The pattern of these thermal effects, based on the molar ratio [CuPaeNs]/[FSCN1], can then be analyzed to derive the thermodynamic parameters of the interaction. During the titration process, a thermal change was observed, indicating a direct interaction between CuPaeNs and FSCN1. The dissociation constant (*K*_d_) was 3.16 µm, and the stoichiometry was 0.950. The *K*_d_ value observed in this study is significantly lower than that reported for the interaction between FSCN1 and NP‐G2‐044 (5–20 µm) in previous research,^[^ ^11^ ^]^ indicating that CuPaeNs bind to FSCN1 more effectively than NP‐G2‐044. In addition, the binding of CuPaeNs with FSCN1 was further visualized using cellular immunofluorescence. As depicted in Figure 4G,H, the red fluorescence‐stained FSCN1 co‐localized with green fluorescence‐labeled Ce6‐CuPaeNs. Since FSCN1's mechanism of action involves binding to F‐actin and cross‐linking F‐actin into dense, rigid bundles, facilitating tumor cell migration and invasion, we confirmed that CuPaeNs strongly bind to FSCN1 to reduce the actin‐bundling activity of FSCN1. As shown in Figure 4I, co‐immunoprecipitation (Co‐IP) analysis in B16 melanoma cells revealed a constitutive interaction between FSCN1 and F‐actin, which was significantly attenuated by CuPaeNs treatment. Additionally, CuPaeNs treatment not only induced morphological changes in B16 cells but also significantly reduced FSCN1 fluorescence intensity (Figure S21, Supporting Information, quantitative analysis from Figures 3B (B16) and S19, Supporting Information (4T1)), implying potential downregulation of FSCN1 expression. To confirm this, we performed Western blotting (WB) analysis (Figure 4J), which validated a markedly reduction in FSCN1 protein levels after CuPaeNs treatment. Importantly, control experiments demonstrated no alterations in β‐actin or F‐actin expression,^[^ ^41^ ^]^ eliminating nonspecific effects on cytoskeletal structural proteins. These data collectively indicate that CuPaeNs specifically suppress FSCN1 expression and impair its actin‐bundling function, ultimately disrupting filopodia formation and suppressing tumor cell migration/invasion.

### 2.5 CuPaeNs Suppressed Tumor Glycolysis via YAP1‐ PFKFB3 Signaling Axis

The Warburg effect drives malignant progression by sustaining elevated glycolysis in tumor cells despite normoxic conditions.^[^ ^42^ ^]^ Fascin, beyond its cytoskeletal functions, critically regulates tumor metabolism through PFKFB3 (6‐phosphofructo‐2‐kinase/fructose‐2, 6‐bisphosphatase 3), which is a key glycolytic enzyme mechanistically linked to YAP1 activation.^[^ ^17^ ^]^ Genetic ablation of fascin via knockdown (KD) or knockout (KO) suppresses the YAP1‐PFKFB3 axis, impairing glycolytic flux in lung adenocarcinoma models.^[^ ^17^ ^]^ Building on our findings that CuPaeNs downregulated fascin expression and disrupted its actin‐bundling activity, we hypothesized that this effect of CuPaeNs might extend to tumor metabolic reprogramming.

**Figure 5:**
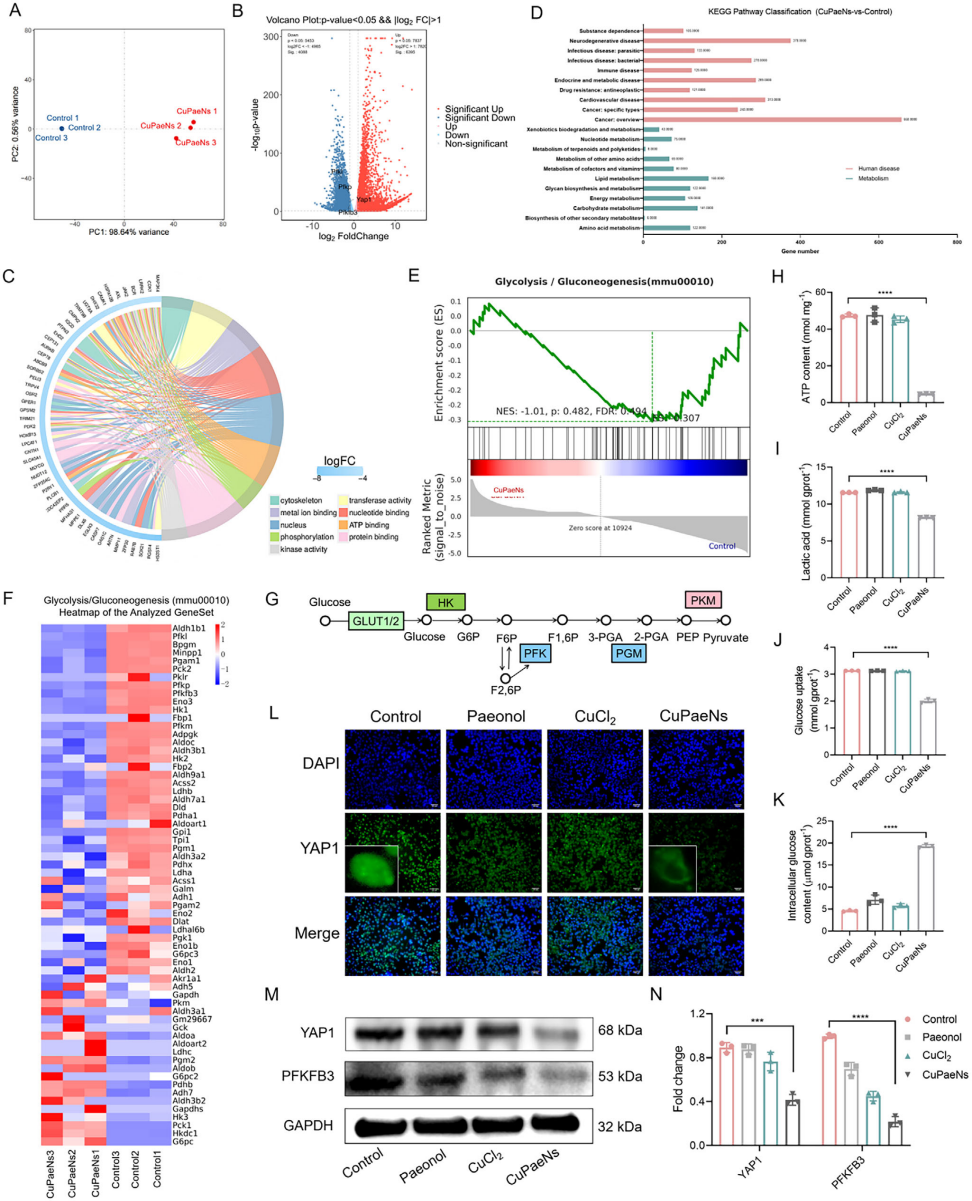
CuPaeNs suppressed the glycolysis via the YAP1‐PFKFB3 regulatory axis. A) Principal component analysis of B16 cells before and after the treatment of CuPaeNs. B) Volcano plots showing the DEGs in the B16 cells before and after the treatment of CuPaeNs. C) GO and D) KEGG analysis of the B16 cells before and after the treatment of CuPaeNs. E) GSEA analysis and F) heatmap of the glycolysis pathway in B16 cells. NES and FDR were indicated. G) Schematic illustrating CuPaeNs inhibition of glycolysis. H–K) Effect of different treatments on ATP content, lactate production, and glucose uptake in B16 cells. (L) Representative immunofluorescence images showing YAP1 subcellular localization in B16 cells with CuPaeNs treatment, scale bar = 100 µm. (m) Protein expression of YAP1 and PFKFB3 within B16 cells in different groups along with (N) quantitative analysis (*n* = 3). Statistically significant differences are indicated with their respective *p*‐values (^***^ *p* < 0.001, and ^****^ *p* < 0.0001).

To investigate the transcriptomic effects of CuPaeNs, we performed RNA‐seq on B16 melanoma cells treated with CuPaeNs vs untreated controls. Principal component analysis (PCA) revealed clear segregation between groups (**Figure** **5**A), with the CuPaeNs‐treated cells clustering distantly from controls (PC1: 98.64% variance). This pronounced divergence in mRNA expression profiles suggested CuPaeNs broadly disrupt transcriptional programs critical for tumor cell proliferation. Using a stringent differential expression threshold (false discovery rate (FDR) < 0.05, |log2 fold change| > 1), we identified 10213 differentially expressed genes (DEGs) in CuPaeNs‐treated B16 cells, with 6395 genes downregulated and 4088 upregulated compared to controls (Figure 5B). To functionally characterize the DEGs, we performed Kyoto Encyclopedia of Genes and Genomes (KEGG) pathway and Gene Ontology (GO) enrichment analyses. GO analysis revealed significant enrichment of protein binding‐associated molecular functions among DEGs in CuPaeNs‐treated B16 cells vs controls (Figure 5C). KEGG pathway analysis of DEGs identified “Cancer: overview” (Human Diseases) and “Carbohydrate metabolism” (Metabolism) as the significantly enriched pathways (Figure 5D). Furthermore, central carbon metabolism was mapped as a third‐tier functional subclass within the “Cancer: overview” pathway hierarchy. Gene set enrichment analysis (GSEA) identified significant downregulation of the central carbon metabolism‐associated cancer pathway (mmu05230) in CuPaeNs‐treated cohorts, demonstrating a normalized enrichment score (NES) of −0.536 (*p* < 0.001; Figure S22A, Supporting Information). Constituent genes within this pathway displayed pronounced expression divergence relative to controls (Figure S22B, Supporting Information). Central carbon metabolism comprises three core pathways: glycolysis, the pentose phosphate pathway, and the tricarboxylic acid (TCA) cycle.^[^ ^43^ ^]^ Analysis of tertiary carbohydrate metabolism pathways identified glycolysis involvement. Subsequent GSEA of this pathway demonstrated suppressed gene expression in CuPaeNs‐treated groups vs controls, with a NES of −0.307 (Figure 5E). Importantly, glycolytic rate‐limiting enzyme genes (*Pfkfb3*, *Pfkl*, *Pfkm*, *Pfkp*) exhibited suppressed expression in CuPaeNs‐treated cohorts, consistent with heatmap visualization of metabolic reprogramming (Figure 5F). This suppression mechanistically corroborates PFK suppression within the glycolysis arm of oncogenic central carbon metabolism signaling networks (Figure 5G). A more comprehensive schematic of the glycolytic signaling pathway network is provided in the appendix (Figure S23, Supporting Information). Mechanistically, the Hippo pathway transcriptional co‐activator YAP1 functions as an actin cytoskeletal tension sensor and glycolytic regulator in tumors.^[^ ^44^ ^]^ GSEA further revealed that the overall gene expression of the Hippo signaling pathway was downregulated (mmu04390; NES = −0.438, *p* < 0.001; Figure S24, Supporting Information). Heatmap analysis of Hippo pathway transcriptomics revealed signature depletion in YAP1 and its related transcriptional targets (Figure S25, Supporting Information). These findings converge with established oncogenic mechanisms, collectively establishing CuPaeNs‐mediated tumor glycolysis suppression via the YAP1‐PFKFB3 regulatory axis.

Subsequently, quantification of glycolytic flux revealed distinct metabolic profiles across treatment groups. CuPaeNs‐treated B16 cells exhibited 90% ATP depletion and 30‐40% lactate reduction vs controls (Figure 5H,I), indicating potent glycolysis suppression. In contrast, CuCl_2_‐ and paeonol‐treated groups maintained ATP and lactate levels comparable to controls. Furthermore, the CuPaeNs‐treated group exhibited 58% lower glucose uptake compared with controls (Figure 5J), while demonstrating 2.3‐fold higher intracellular glucose accumulation (Figure 5K). These results indicated that CuPaeNs impaired glycolytic flux, allowing transient glucose import but preventing effective catabolism, thereby trapping glucose within cells. Importantly, immunofluorescence analysis demonstrated an significant reduction in YAP1 fluorescence intensity (Figure 5L) with concomitant suppression of its nuclear localization. WB analyses further revealed the suppression of both YAP1 and PFKFB3 (53% for YAP1, 78% for PFKFB3) in B16 cells compared to vehicle controls (Figure 5M,N). The downregulation of PFKFB3, a critical enzyme synthesizing fructose‐2,6‐bisphosphate (F2,6BP) to activate PFK1, directly compromises the committed step of glycolysis. We further assessed the expression of *Yap1* and *Pfkfb3* by quantitative polymerase chain reaction (qPCR). Consistent with the RNA sequencing results, both genes were significantly downregulated in B16 cells treated with CuPaeNs (Figure S26, Supporting Information). Mechanistically, CuPaeNs disrupt FSCN1/F‐actin bundling, thereby suppressing glycolysis via inhibition of the fascin‐YAP1‐PFKFB3 axis. This multimodal action establishes metal‐phenol coordination nanozymes as precision therapeutics capable of concurrently targeting cytoskeletal remodeling to reprogram energy metabolism in tumor cells.

### 2.6 CuPaeNs Suppressed B16 Tumor Growth in Murine Models

Prior to evaluating the in vivo antitumor efficacy, we systematically assessed the biocompatibility of CuPaeNs. As shown in Figures S27 and S28 (Supporting Information), CuPaeNs exhibited negligible cytotoxicity against normal human hepatocytes (L02 cell line), with hemolysis rates maintained below 12% even at 500 µg mL^−1^. Subsequent biosafety studies in healthy mice administered CuPaeNs (50 mg kg^−1^) revealed no abnormalities in hematological parameters at 7‐ and 14‐day post‐injection (Figure S29, Supporting Information). Histological analysis of Hematoxylin and Eosin (H&E)‐stained heart, liver, spleen, lung, and kidney sections confirmed the absence of tissue damage in CuPaeNs‐treated groups (Figure S30, Supporting Information). The biodistribution of CuPaeNs in B16 tumor‐bearing mice was evaluated using both in vivo fluorescence imaging and inductively coupled plasma mass spectrometry (ICP‐MS). As shown in Figure S31 (Supporting Information), CuPaeNs were initially distributed (1 h post‐injection) across multiple organs and tissues, including the lungs, liver, kidneys, and B16 xenografts. Then, we monitored the temporal distribution profiles of CuPaeNs, particularly within lung and tumor sites. Quantitative ICP‐MS analysis revealed a progressive clearance of CuPaeNs from lung tissue over time. Notably, increased retention of CuPaeNs was observed at tumor sites at 6 h post‐injection compared to that at 1 h, followed by a gradual decline thereafter. Together, these data demonstrate the favorable biocompatibility and tumor accumulation capability of CuPaeNs within the tested dosage range.

**Figure 6:**
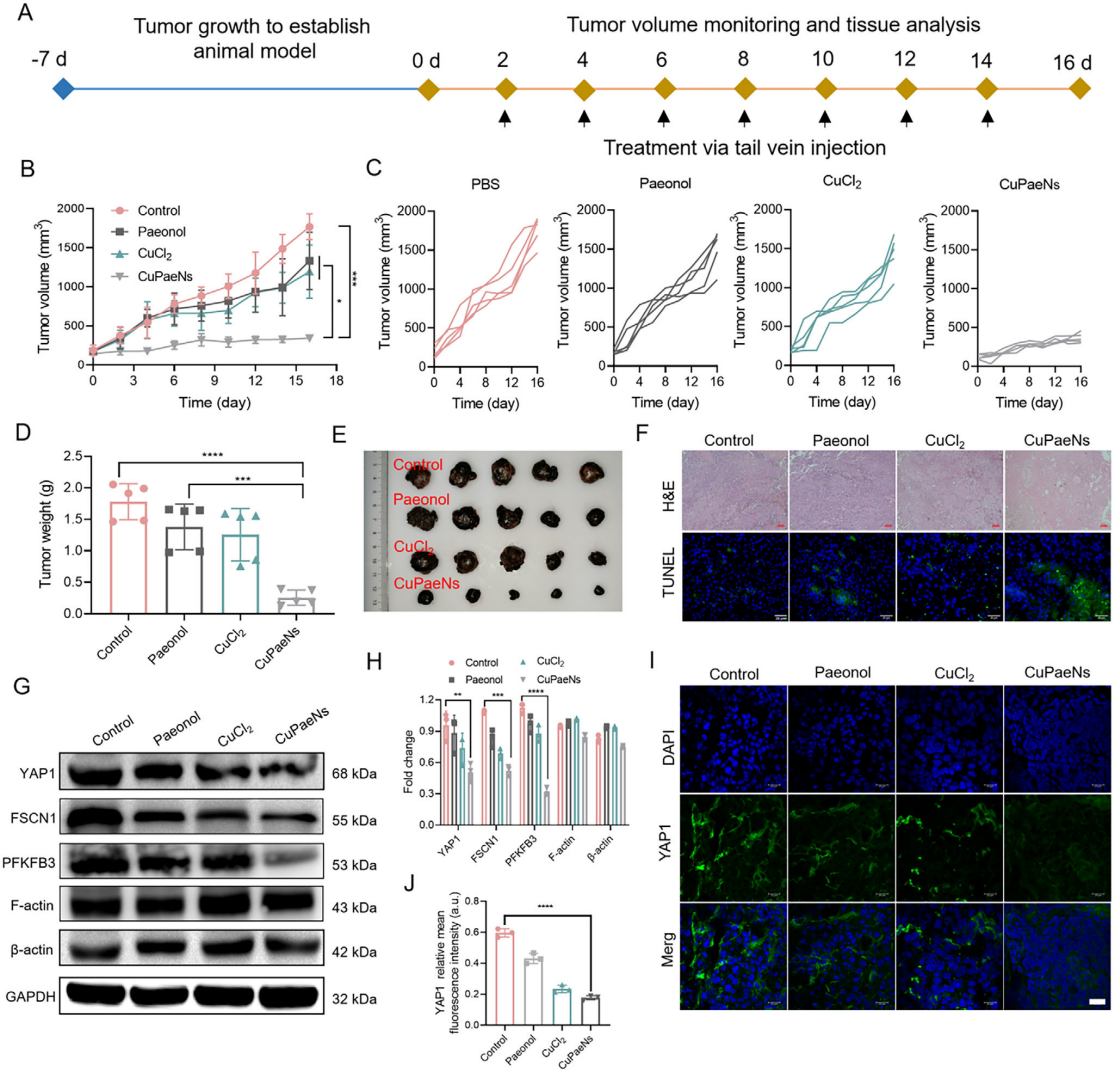
CuPaeNs showed excellent anti‐tumor effect in vivo. A) Schematic diagram depicting tumor inoculation and treatment procedures for B16‐bearing mice. B) B16 tumor growth curves in different groups (*n* = 5). C) Tumor growth curves of individual mice. D) Tumor weights and E) representative images of harvested B16 tumors following treatment with PBS, paeonol, CuCl_2_, and CuPaeNs, respectively. F) Representative H&E stained sections (scale bar = 100 µm) and TUNEL staining (scale bar = 20 µm) of B16 tumor tissue at the end of the experiments. G) Protein expression of YAP1, FSCN1, PFKFB3, F‐actin, and β‐actin at tumors sites in different groups and H) corresponding quantification (*n* = 3). I) Immunofluorescence images (scale bar = 20 µm) and J) corresponding quantification (*n* = 3) showing the expression of YAP1 in tumors treated with PBS, paeonol, CuCl_2_, and CuPaeNs, respectively. Statistically significant differences are indicated with their respective *p*‐values (^*^ *p* < 0.05, ^**^ *p* < 0.01, ^***^ *p* < 0.001, and ^****^ *p* < 0.0001).

Next, the antitumor performance of CuPaeNs in vivo was assessed by setting a B16 tumor‐bearing mice model. As demonstrated in **Figure** **6**A, the mice were intravenously injected with PBS, paeonol, CuCl_2_, or CuPaeNs on days 2, 4, 6, 8, 10, 12, 14, and the tumor growth of mice was monitored for 16 days. The injection dose of CuPaeNs was 25.0 mg kg^−1^, and the CuCl_2_ and paeonol were fixed at an equivalent concentration of CuPaeNs. After 16 days of treatment, the therapeutic efficacy over time was assessed by monitoring tumor volumes in the four experimental groups (Figure 6B). Compared to the PBS group, paeonol and CuCl_2_ alone minimally inhibited tumor growth, with all tumors in these two groups reaching 1300 and 1200 mm^3^ within 16 days, respectively. In contrast, the administration of CuPaeNs markedly suppressed tumor growth, resulting in an average tumor volume of 340 mm^3^. This indicated that treatment with paeonol or CuCl_2_ alone had a less potent therapeutic effect, whereas the nanozymes formed by Cu^2+^ and paeonol resulted in a substantial suppression of tumor growth. Additionally, we assessed the influence of individual differences within each group (Figure 6C), and all mice in the groups displayed similar tumor growth curves. Moreover, the body weight of mice in all four groups did not show significant changes during the 16‐day treatment period (Figure S32, Supporting Information). At the end of the treatment protocol, all mice were euthanized, and ex vivo tumor weights were measured and presented in Figure 6D,E. The results showed that CuPaeNs achieved an 86% tumor growth inhibition rate, whereas paeonol and CuCl_2_ had inhibition rates of 23% and 29%, respectively. Histological analysis further revealed that the use of paeonol or CuCl_2_ alone had a limited impact on B16 tumors, as evidenced by small areas of necrosis observed in H&E stained sections or a small proportion of apoptotic cells detected by fluorescent terminal deoxynucleotidyl transferase dUTP nick end labeling (TUNEL) staining (Figure 6F). In contrast, treatment with CuPaeNs led to extensive necrosis and apoptosis. Furthermore, no significant abnormalities in major organs were observed during the treatments (Figure S33, Supporting Information). To further confirm the antitumor mechanism of CuPaeNs through glycolysis suppression, the corresponding protein expression was analysis by WB analysis. The results in Figure 6G,H showed that CuPaeNs significantly reduced the levels of PFKFB3 and YAP1 in tumor slices from B16‐bearing mice. Given that fascin expression strongly correlates with PFKFB3 and YAP1 target genes, the decreased expression of FSCN1 was also confirmed. Additionally, the tumor slices were further stained for YAP1 and FSCN1. As depicted in Figures 6I,J and S34 (Supporting Information), treatment with CuPaeNs notably decreased the protein levels of FSCN1 in the tumor sections, leading to the inhibition of YAP1 expression compared to treatment with CuCl_2_ and paeonol alone. To further evaluate the in vivo therapeutic outcome of CuPaeNs, another breast tumor model was established in BALB/c mice using 4T1 cells. As shown in Figure S35 (Supporting Information), CuPaeNs significantly suppressed the tumor growth, mirroring the performances observed in B16‐bearing mice. These findings provide direct evidence of CuPaeNs impede tumor growth via the YAP1‐PFKFB3 pathway.

**Figure 7:**
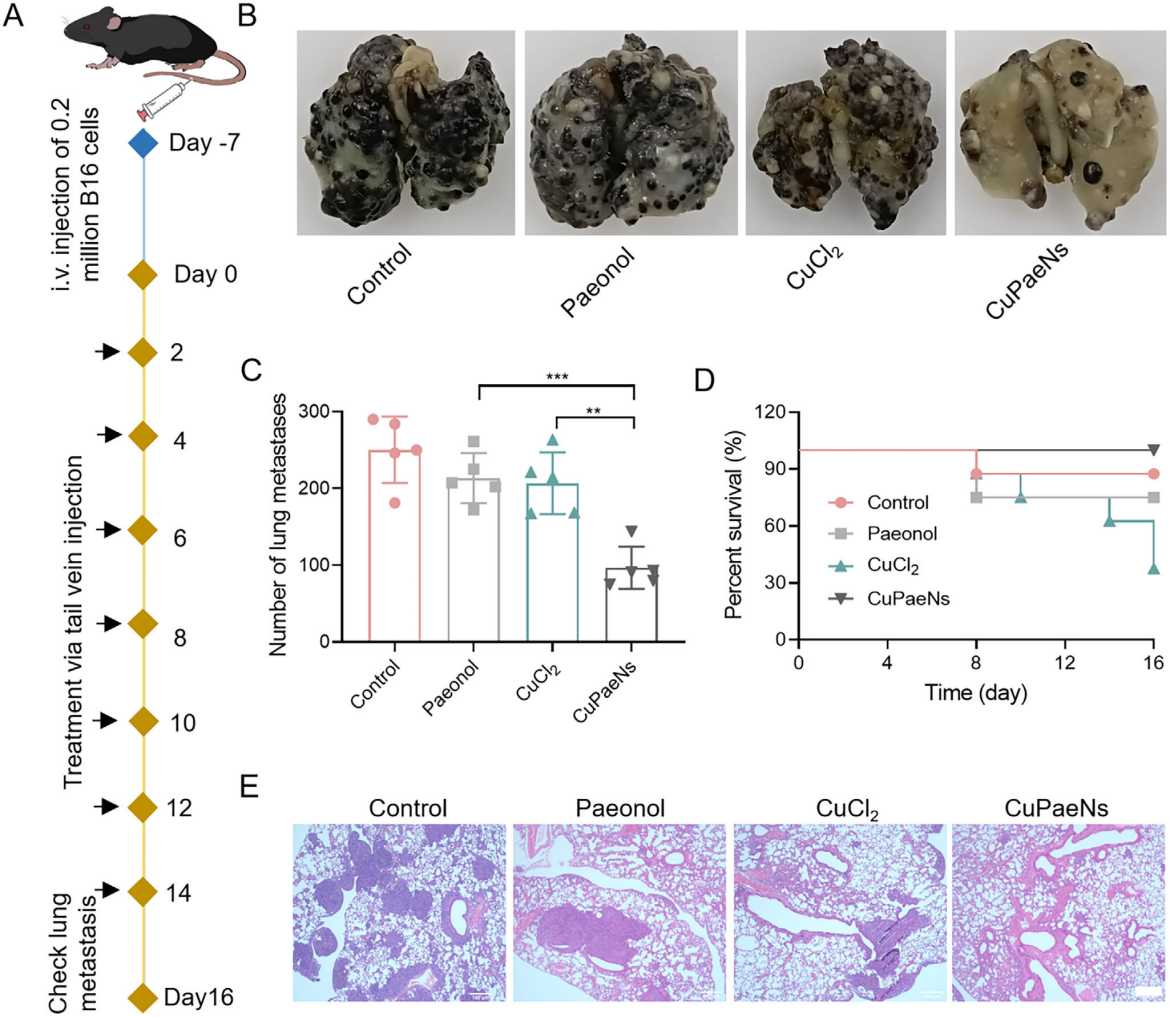
CuPaeNs possessed anti‐metastasis capacity in vivo. A) Schematic diagram depicting the B16‐bearing mice employed to investigate the impact of CuPaeNs on lung metastasis. B) Photographs of lungs collected on day 16 after i.v. injection of B16 cells following various treatments. C) Counting tumor nodules in the lungs following different treatments. D) Survival curves of B16‐bearing mice following various treatments. E) H&E staining of the lung tissues collected on day 16, scale bar = 100 µm. Statistically significant differences are indicated with their respective *p*‐values (^**^ *p* < 0.01, ^***^ *p* < 0.001).

To further investigate the therapeutic efficacy against challenging tumors, we assessed the antimetastatic potential of CuPaeNs. B16 cells were intravenously injected into mice to simulate tumor cell metastasis. Subsequently, mice received intravenous injections of saline, paeonol, CuCl_2_, or CuPaeNs on days 2, 4, 6, 8, 12, and 14 (**Figure** **7**A). At the end of the protocol, lung specimens were collected from each group to assess the extent of tumor metastasis. Analysis of lung photographs revealed a profound suppression of lung metastasis in mice treated with CuPaeNs (Figure 7B). The number of metastatic lesions in the lungs of CuPaeNs‐treated mice was markedly lower compared to other groups (Figure 7C), and all B16 tumor‐bearing mice in the CuPaeNs‐treated group survived (Figure 7D). Conversely, mice in other groups, including those treated with CuCl_2_ and paeonol, experienced partial mortality. Furthermore, histological examination using H&E staining (Figure 7E) showed a significant reduction in the area of dense tissue indicative of tumor metastasis following CuPaeNs treatment. These findings underscore the promising anti‐metastatic efficacy of CuPaeNs, attributed to its potent ability to disrupt fascin's actin‐bundling activity.

## 3 Discussion

Most current treatments for metastatic tumors primarily aim to eliminate or halt the growth of primary and metastatic tumor cells. Despite the significance of tumor cell migration and invasion in the metastatic process, specific drugs targeting these processes have not yet been integrated into standard metastatic tumor treatments. Therefore, there is a compelling need to develop high‐performance drugs that specifically target tumor cell migration and invasion as novel approaches for treating metastatic tumors. Fascin, a pro‐metastatic actin‐bundling protein upregulated in metastatic carcinomas, promotes tumor cell migration and invasion by facilitating membrane protrusions. Our study provides promising insights into the potential benefits of CuPaeNs, a nanozyme, inhibiting tumor growth by catalytically amplifying TME oxidative stress. Importantly, we also reveal that CuPaeNs block fascin‐actin bundling to prevent the formation of dense and rigid bundles crucial for generating finger‐like processes that facilitate tumor cell migration and invasion. Furthermore, we found that CuPaeNs could further suppress glycolysis by modulating the fascin‐YAP1‐PFKFB3 axis. Importantly, CuPaeNs demonstrated robust antitumor efficacy in murine models of both melanoma and breast tumor. Collectively, this metal‐phenolic nanozyme targets tumor‐specific ROS generation and disrupts fascin‐mediated actin bundling, effectively suppressing tumor growth and metastatic colonization, especially in malignancies with fascin overexpression.

## 4 Experimental Section

### Materials

Crystal violet and copper (II) chloride dihydrate were obtained from BBI Life Sciences (Hong Kong, China). EDTA‐2Na and regenerated cellulose membrane tubing were sourced from Shanghai Yuanye Bio‐Technology Co., Ltd (Shanghai, China). 2′‐Hydroxy‐4′‐methoxyacetophenone was procured from Adamas‐beta (Shanghai, China). Dulbecco's Modified Eagle′s Medium (DMEM) and RPMI Medium Modified were purchased from Cytiva (USA). Thiazolyl Blue Tetrazolium Bromide (MTT), PrEST antigen FSCN1, and polyvinylpyrrolidone were acquired from Sigma‐Aldrich (USA). Actin‐Tracker Red‐Rhodamine, DAPI staining solution, trizol, dimethylsulfoxide (DMSO, 99.9%), apoptosis and necrosis assay kit, ATP assay kit, mitochondrial membrane potential assay kit with 5,5′,6,6′‐tetrachloro‐1,1′,3,3′‐tetraethyl‐imidacarbocyanine iodide (JC‐1), β‐actin mouse monoclonal antibody (1:1000), PFKFB3 rabbit polyclonal antibody (1:1000), GAPDH mouse monoclonal antibody (1:1000), were obtained from Beyotime Biotechnology (Shanghai, China). Fascin polyclonal antibody (1:800) was purchased from Proteintech (Wuhan, China). Mouse Control IgG was purchased from ABclonal (Guangzhou, China). Anti‐F‐actin antibody (1:1000) was obtained from Abcam (UK). Lactic acid (LA) colorimetric assay kit and glucose (GLU) fluorometric assay kit were purchased from Elabscience (USA). YAP1 Rabbit (1:1000) was obtained from Cell Signaling Technology, Inc (USA). Annexin V fluorescein isothiocyanate (Annexin V‐FITC) apoptosis assay kit was ordered from Shanghai Universal Biotech Co., Ltd (Shanghai, China).

### Preparation of CuPaeNs

First, 66 mg of PVP was dissolved in 5 mL of ethanol, and 20 mg of CuCl_2_·2H_2_O in 1 mL of ethanol was added dropwise while stirring. After 1 min, 40 mg of paeonol in 1 mL of ethanol was added dropwise, followed by 4 h of stirring. Subsequently, the products were dialyzed against water overnight for further use. The synthesis of MgPae, MnPae, CaPae, and CoPae followed a similar method with the same metal‐to‐paeonol ratio.

### Cell Viability Assay

B16, 4T1, CT26, K7M2, and SiHa cells were plated in 96‐well plates at a density of 5 × 10^3^ cells/well and incubated for 24 h. The cytotoxicity of CuPaeNs against these tumor cells was evaluated using an MTT assay after treating with varying concentrations of CuPaeNs. The concentrations of CuCl_2_ and paeonol were adjusted to match their respective amounts in CuPaeNs. To further evaluate the apoptotic efficacy of CuPaeNs, B16 cells were plated in 24‐well plates at a density of 2 × 10^4^ cells /well and incubated for 24 h. Subsequently, the B16 cells were treated with PBS, CuCl_2_, paeonol, and CuPaeNs. After 24 h, the cells were stained with Hoechst and PI for 20 min, washed with PBS, and fluorescence images were acquired using CLSM. Additionally, apoptosis was assessed using flow cytometry with Annexin V‐FITC and PI staining. In a separate experiment, B16 cells were seeded in 6‐well plates at a density of 1 × 10^5^ cells/well and cultured for 24 h. They were then treated with PBS, CuCl_2_, paeonol, and CuPaeNs, followed by staining with Annexin V‐FITC and PI, and apoptosis was quantified by flow cytometry.

### High‐Content Image Acquisition and Analysis

B16 cells (4×10^3^ cells/well) were seeded in 96‐well plates. After incubation at 37 °C for 24 h, the cells were treated with PBS, paeonol, CuCl_2_, and CuPaeNs, respectively, and transferred to the PerkinElmer Operetta CLS High‐Content Imaging System (PerkinElmer, USA) for an additional 24 h of observation. The system automatically tracked cell division and proliferation, with data collection and analysis performed using Harmony 4.1 software.

### Isothermal Titration Calorimetry

Calorimetric measurements were performed using a Low Volume Nano ITC instrument from TA Instruments. In each trial, CuPaeNs solution was titrated into the protein solution within a 350 µL sample cell, while the reference cell contained 0.1 mm sodium azide in double‐distilled water. All measurements were conducted at 25 °C. Experimental heats from protein‐inhibitor titrations were corrected for dilution heats by subtracting the average heat of the last three measurements post‐saturation. Each measurement was conducted in triplicate at minimum.

### Western Blotting Analysis

To assess protein expression levels associated with fascin, B16 cells were initially seeded in 60 mm × 15 mm cell culture dishes and incubated for 24 h. The B16 cells were subsequently treated with PBS, CuCl_2_, paeonol, and CuPaeNs. Cell lysates were prepared and subjected to sodium dodecyl sulfate polyacrylamide gel electrophoresis (SDS‐PAGE) for analysis, followed by quantification of protein expression levels using ImageJ software.

### Co‐Immunoprecipitation Experiments

The cells were plated in 60 mm × 15 mm cell culture dishes and incubated for 24 h. Subsequently, the cells were treated either with CuPaeNs. Following centrifugation at 15000 × g for 10 min at 4 °C, the supernatant was collected and incubated overnight at 4 °C with the respective primary antibody. Protein G Sepharose beads were added and incubated further at 4 °C, the beads were washed three times with PBS, then boiled for 10 min in 100 µL of 5 × SDS loading buffer.

### Transcriptomics Analysis

The cellular RNA was extracted using Trizol reagent following the manufacturer's protocol. RNA purity and quantity were assessed using the NanoDrop 2000 spectrophotometer, while RNA integrity was evaluated using the Agilent 2100 Bioanalyzer. Subsequently, libraries were prepared using the VAHTS Universal V6 RNA‐seq Library Prep Kit according to the manufacturer's instructions. Transcriptome sequencing and analysis were conducted by OE Biotech Co., Ltd. (Shanghai, China).

### In Vivo Anti‐Melanoma Efficacy of CuPaeNs

All animal experiments were conducted following approved protocols by the Animal Care Committee of Yangzhou University (ethical approval number: YXYLL‐2021‐16). Male BALB/c mice (four‐week‐old) were obtained from Yangzhou University and used in this study. To establish a B16‐bearing animal model, 100 µL of B16 cells (5 × 10^6^ cells) suspended in RPMI 1640 were subcutaneously injected into mice. Once tumors reached ≈100 mm^3^, mice were divided into 4 groups (*n* = 5): I) control group, intravenous (i.v.) injection of PBS, II) i.v. injection of CuCl_2_, III) i.v. injection of paeonol, and IV) i.v. injection of CuPaeNs (25 mg kg^−1^). Dosages of CuCl_2_ and paeonol were adjusted to match equivalent contents in CuPaeNs. Each group received a single dose every two days. Throughout treatment, body weights and tumor volumes were monitored using the formula V = L × W^2^ / 2 (where W is the minimum diameter and L is the maximum diameter). On day 16, mice were euthanized. The tumor tissues and major organs were collected for further analysis. The assay of the 4T1‐bearing model followed a similar method and treatment.

### In Vivo Anti‐Melanoma Lung Metastasis Efficacy of CuPaeNs

Six‐week‐old male C57BL/6 mice were used to establish a B16‐induced lung metastasis model by i.v. injecting 100 µL of 5 × 10^5^ B16 cells. One week later, the mice were then divided into 4 groups (*n* = 5): I) control group, i.v. injection of PBS, II) i.v. injection of CuCl_2_, III) i.v. injection of paeonol, and IV) i.v. injection of CuPaeNs. Each group received a single dose every two days. Throughout treatment, the body weights of the mice were monitored. On day 16, the mice were euthanized, and lung tissues were collected for further analysis.

### Statistical Analysis

The data were presented as mean ± standard deviation. Statistical significance was evaluated using Student's t‐test for pairwise comparisons, as implemented in GraphPad Prism 8.0 software. Statistical significance levels are denoted by asterisks as follows: ^*^ *p* < 0.05, ^**^ *p* < 0.01, ^***^ *p* < 0.001, and ^****^ *p* < 0.0001.

## Conflict of Interest

The authors declare no conflict of interest.

## Author Contributions

J.X., L.G., and H.D. led this protect. P.Z. designed the experiments, analyzed the data, and prepared the manuscript. Y.W., J.X., and H.L. conducted the experiments, analyzed the data, and participated in the writing of the manuscript. S.Z. and L.F. analyzed the data and discussed the manuscript. All authors commented on the manuscript.

## Supporting information



Supporting Information

Supplemental Video 1

Supplemental Video 2

Supplemental Video 3

Supplemental Video 4

## Data Availability

The data that support the findings of this study are available from the corresponding author upon reasonable request.
