# Supplementary material for: Beyond Catalytic Therapy: Copper‐Paeonol Nanozymes Disrupt Fascin‐Mediated Actin Bundling to Suppress Tumor Growth and Metastasis
Source: Adv Sci (Weinh). 2025 Dec 17;13(18):e12186. doi: 10.1002/advs.202512186 (PMC13042531; doi:10.1002/advs.202512186)
Supplement: Supplementary file 1 — Supporting Information [file ADVS-13-e12186-s005.docx]

Supporting Information

**Beyond Catalytic Therapy: Copper-Paeonol Nanozymes Disrupt Fascin-Mediated Actin Bundling to Suppress Tumor Growth and Metastasis**

Peiying Zhang^a,b^, Huajun Li^a^, Yisen Wang^a^, Jie Xiang^a^, Lei Fan^c^, Shengzhe Zhang^a^, Lizeng Gao^d,^*, Hua Dai^a,^*, Juqun Xi^a,b,^*

^a^ School of Traditional Chinese Medicine, Faculty of Medicine, Yangzhou University, Yangzhou, Jiangsu 225009, China.

^b^ Key Laboratory of the Jiangsu Higher Education Institutions for Integrated Traditional Chinese and Western Medicine in Senile Diseases Control (Yangzhou University), Yangzhou, Jiangsu 225009, China.

^c^ School of Chemistry and Materials, Yangzhou University, Yangzhou, Jiangsu 225002, China.

^d^ CAS Engineering Laboratory for Nanozyme, Key Laboratory of Biomacromolecules, Institute of Biophysics, Chinese Academy of Sciences, Beijing 100101, China.

*Corresponding authors: gaolizeng@ibp.ac.cn (L.Gao), daihua@yzu.edu.cn (H. Dai), and xijq@yzu.edu.cn (J. Xi)

**METHODS**

**Characterization**

The morphology, composition and structure of CuPaeNs were observed and characterized by various techniques, including Tecnai 12 transmission electron microscope (TEM, Philips), Tecnai G2 F30 S-TWIN Field emission transmission electron microscopy (FETEM, FEI), D8 ADVANCE X-ray diffractometer (XRD, Bruker), ESCALAB 250Xi X-ray electron spectrometer (XPS, Thermo Scientific), Nicolet iS20 Fourier transform infrared spectroscopy (FTIR, Thermo Scientific) and P1 ultraviolet-visible spectrophotometer (UV, MAPADA). The hydrodynamic diameter and zeta potential of CuPaeNs were determined by dynamic light scattering (DLS, Malvern). The content of Cu^2+^ in CuPaeNs was determined by Optima 7300DV inductively coupled plasma atomic emission spectrometer (ICP-AES, PerkinElmer). Gemini SEM 300 confocal laser scanning microscopy (CLSM, Carl Zeiss) was adopted to observe the signals of fluorescence probes.

**Catalytic activity detection**

To determine whether CuPaeNs show peroxidase-like activity, 0.5 mg mL^-1^ CuPaeNs was added into 0.1 M HAc-NaAc buffer solution (pH 4.5, total volume: 200 μL) containing TMB (final concentration: 0.832 mM) and H_2_O_2_ (300 mM). The UV-vis absorbance spectra of oxidized TMB were recorded via a microplate reader. pH dependence assay of the catalytic activity was carried out by employing 0.5 mg mL^-1^ CuPaeNs in different pH buffers (1.0, 2.0, 3.0, 4.0, 4.5, 5.0, 6.0, 7.0, 8.0, 9.0, 10.0). The steady-state kinetic assays of CuPaeNs with H_2_O_2_ as the substrate were performed by adding nanoparticles 0.5 mg mL^-1^ into 0.1 M HAc-NaAc buffer solution (pH 4.5, total volume: 200 μL) containing TMB (final concentration: 0.832 mM) and different concentrations of H_2_O_2_ (0, 18.75, 37.5, 75, 150, 300 mM).

**Measurement of mitochondrial membrane potential**

The change of mitochondrial membrane potential was assessed using JC-1 as a fluorescent probe. B16 cells were seeded in 6-well plates at a density of 1 × 10^5^ cells per well and cultured for 24 h. Subsequently, the cells were treated with PBS, CuCl_2_, paeonol and CuPaeNs. After treatment, the cells were washed with PBS and stained with JC-1 dye. Fluorescence images of the cells were captured using a fluorescence microscope.

**Detection of GSH and GSSG**

B16 cells (1.2 × 10^5^ ) were seeded on 6-well plate (Nest). CuPaeNs were added to cells for 24 hours. And the cells with CuPaeNs and without were washed with PBS and colleted using Biochemical assay regent (Elabscience, C: E-BC-K097-M) according to the manufacturer's protocol.

**Wound healing assay**

B16 cells were seeded into 6-well plates at a density of 2 × 10^5^ cells per well. Once the cell density had reached 80-90%, the cell monolayer in each well was rapidly scratched with a 200 μL pipet tip. Subsequently, DMEM containing PBS, CuCl_2_, paeonol, or CuPaeNs was added and incubated for 24 h. Microscopy was employed to record the cell scratch closure.

**Cellular uptake behavior**

CuPaeNs were first labeled by chlorine6 (Ce6) to prepare Ce6-CuPaeNs. B16 (2 × 10^6^ cells/well) were seeded in a CLSM-specific dish for CLSM analysis. When the density reached ~ 80%, Ce6-CuPaeNs were added, and the cells were cultured at 37 °C in a humidified incubator for another 3 h, 6 h, 9 h and 12 h. Then, the cells were washed with PBS three times, and the nuclei were stained with DAPI. A CLSM unit was utilized to obtain the fluorescence images of the cells. Additionally, the B16 cell treated CuPaeNs for 5 h were collected and treated with electron microscope fixative. Then the cells were transferred to copper mesh and photographed by TEM.

**Cell invasion assay**

Matrigel gel-lined cell chambers were situated in 12-well plates, after which 100 μL of B16 cells (1×10^6^ cells/well) resuspended in serum-free medium was added to the top chamber. Following that, 600 μL of medium containing 15% fetal bovine serum (FBS) was supplemented to the bottom chamber, and subsequently, the cells were treated with PBS, CuCl_2_, paeonol and CuPaeNs. After a 24 h incubation period, the non-invaded cells in the top chamber (containing matrigel) were extracted and fixed with paraformaldehyde. The cells were later stained with 0.5% crystalline violet for 20 min and visualized under a microscope.

**Fluorescence microscopy**

B16 cells were seeded onto laminin-coated glass coverslips for 24 h with or without CuPaeNs (250 μg mL^-1^). Cells were fixed with 3.7% formaldehyde in PBS for 10 min at room temperature, permeabilized with 0.1% Triton X-100 for 5 min, and then washed three times with PBS. Anti-fascin monoclonal antibody, actin-tracker red-rhodamine and DAPI were used for immunofluorescent staining. The coverslips were then mounted onto slides and imaged using Zeiss confocal microscopy.

**Molecular docking**

Molecular docking was performed using Autodock vina with the default docking method and running on Windows. The chemical structure of paeonol was obtained from the Pubchem compound database with PubChem CID as 11092. The three-dimensional structure of human FSCN1 was retrieved from the Protein Data Bank (ID: 3LLP) and obtained from the Research Collaboratory for Structural Bioinformatics (RCSB) Protein Data Bank. The molecular composition of a crystalline unit is constructed, and then use the function of align in pymol to construct a multicellular structure. The grid box was generated to cover the entire receptor with the receptor center as the grid center. Gasteiger atomic partial charges were assigned for all investigated ligands. The docking results were analyzed and represented using PyMOL.

**Co-localization assay**

B16 cells were seeded onto laminin-coated glass coverslips for 24 h with or without Ce6-CuPaeNs (250 μg mL^-1^). Cells were fixed with 3.7% formaldehyde in PBS for 10 min at room temperature, permeabilized with 0.1% Triton X-100 for 5 min, and then washed three times with PBS. Anti-fascin monoclonal antibody and DAPI were used for immunofluorescent staining. The coverslips were then mounted onto slides and imaged using Zeiss confocal microscopy.

**Lysosomal/** **CuPaeNs colocalization**

B16 cells (1.2 × 10^5^) were seeded on glass-bottom dishes (Nest). Rhodamine-labeled CuPaeNs were added to cells. After incubation for 3, 6 and 9 hours, the treated cells were washed with PBS. The cells were stained with Lysotracker Green (dilution ratio 1:14000) (Beyotime, C1047S) for 1 hour at 37°C and 4′,6-diamidino-2-phenylindole (DAPI) (Beyotime, C1006) for 10 min at room temperature. The confocal images were obtained from Olympus FV3000 (Japan), and PCC analysis was performed using ImageJ.

**Determination of ATP content**

B16 cells (1 × 10^5^ cells/well) were seeded in 6-well plates and cultured for 24 h. They were then treated with PBS, CuCl_2_, paeonol and CuPaeNs. After treatment, B16 cells were harvested and the supernatant was separated by mixing it with 200 μL of lysate. The quantity of ATP in the supernatant was estimated via an enhanced ATP assay kit.

**Detection of glucose content in B16 cells**

B16 cells were seeded in 6-well plates at a density of 1 × 10^6^ cells per well and grown in 5% CO_2_ at 37 ℃ overnight. Subsequently, the cells were treated with PBS, CuCl_2_, paeonol and CuPaeNs. After treatment, the cell medium was collected and B16 cells were harvested and the supernatant was separated by mixing it with 200 μL of lysate. Both of them were measured by employing a glucose (GLU) fluorometric assay kit. The glucose fluorometric assay kit was carried out according to the manufacturer’s instructions.

**Detection of lactic acid content in B16 cells**

B16 cells were seeded in 6-well plates at a density of 1 × 10^6^ cells per well and grown in 5% CO_2_ at 37℃ overnight. Subsequently, the cells were treated with PBS, CuCl_2_, paeonol and CuPaeNs. After treatment, the cell medium was collected and B16 cells were harvested and the supernatant was separated by mixing it with 200 μL of lysate. Both of them were measured by employing a lactic acid (LA) colorimetric assay. The L-lactic acid (LA) colorimetric assay kit was carried out according to the manufacturer’s instructions.

**Biosafety analysis**

L02 cells were added to 96-well plates at a density of 5 × 10^3^ cells per well and cultured for 24 h. After the addition of varying concentrations of CuPaeNs, the toxicity of CuPaeNs towards normal cells was assessed using an MTT assay. Healthy 5-week-old BALB/c male mice were randomly assigned to three groups (*n* = 3) and subsequently intravenous injected with PBS and CuPaeNs (50 mg kg^-1^), respectively. On day 7 and day 14, blood was collected via ocular puncture from each group of mice, and the collected serum and whole blood underwent regular blood analysis and biochemical indexing. The heart, liver, spleen, lung, and kidney of each group of mice were collected and subjected to H&E staining as well as microscopic observation and photography.

***In vivo* biodistribution assay**

B16 melanoma-bearing mice were randomly assigned to experimental groups (*n* = 3 per time point) and intravenously injected with CuPaeNs at a dose of 25 mg kg⁻¹ body weight (100 μL per mouse, formulated in sterile phosphate-buffered saline (PBS, pH 7.4)). At predetermined time intervals (1, 6, 12, 24, and 48 h post-injection), mice were euthanized and major organs (heart, liver, spleen, lung, kidneys) as well as tumor tissues were collected and digested with nitric acid at 120 °C to obtain clear solutions, which were then used for biodistribution detection using NexION 2000 (PerkinElmer).

**Quantitative real-time PCR (qPCR) analysis**

Total RNA was extracted from cells using FastPure Cell/Tissue Total RNA Isolation Kit V2 (Vazyme, C:RC112-01) according to the manufacturer's protocol. The concentration and purity of RNA were determined using a NanoDrop 2000 spectrophotometer (Thermo Fisher Scientific), with A260/A280 ratios between 1.8 and 2.0 considered acceptable. Complementary DNA (cDNA) was synthesized from 1 μg of total RNA using a PrimeScript RT reagent kit with gDNA Eraser (Takara), following the manufacturer's instructions. qPCR was performed on a StepOnePlus Real-Time PCR System (Applied Biosystems) using SYBR Premix Ex Taq II (Takara). The reaction mixture (20 μL total volume) contained 10 μL of 2× SYBR Premix, 0.4 μL of each forward and reverse primer (10 μM), 2 μL of cDNA template (1:10 dilution), and 7.2 μL of nuclease-free water. The thermal cycling conditions were as follows: initial denaturation at 95°C for 30 s, followed by 40 cycles of denaturation at 95°C for 5 s, and annealing/extension at 60°C for 30 s. A melting curve analysis was performed after amplification to verify the specificity of PCR products, with conditions: 95°C for 15 s, 60°C for 1 min, and gradual heating to 95°C. β-actin was used as the housekeeping gene for normalization. The relative gene expression levels were calculated using the 2^(-ΔΔCt) method. The sequences of primers are in the Table S2.


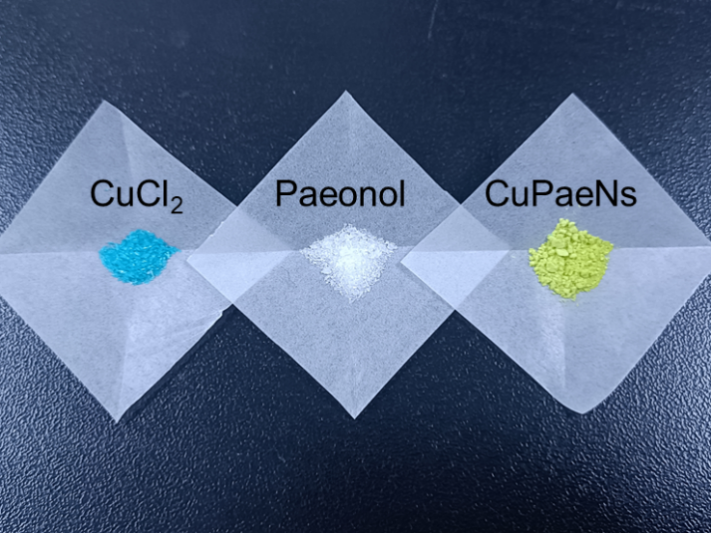


**Figure S1.** Color change photographs of CuCl_2_, paeonol, and CuPaeNs.

**Figure S2.** XRD spectrum of CuPaeNs.


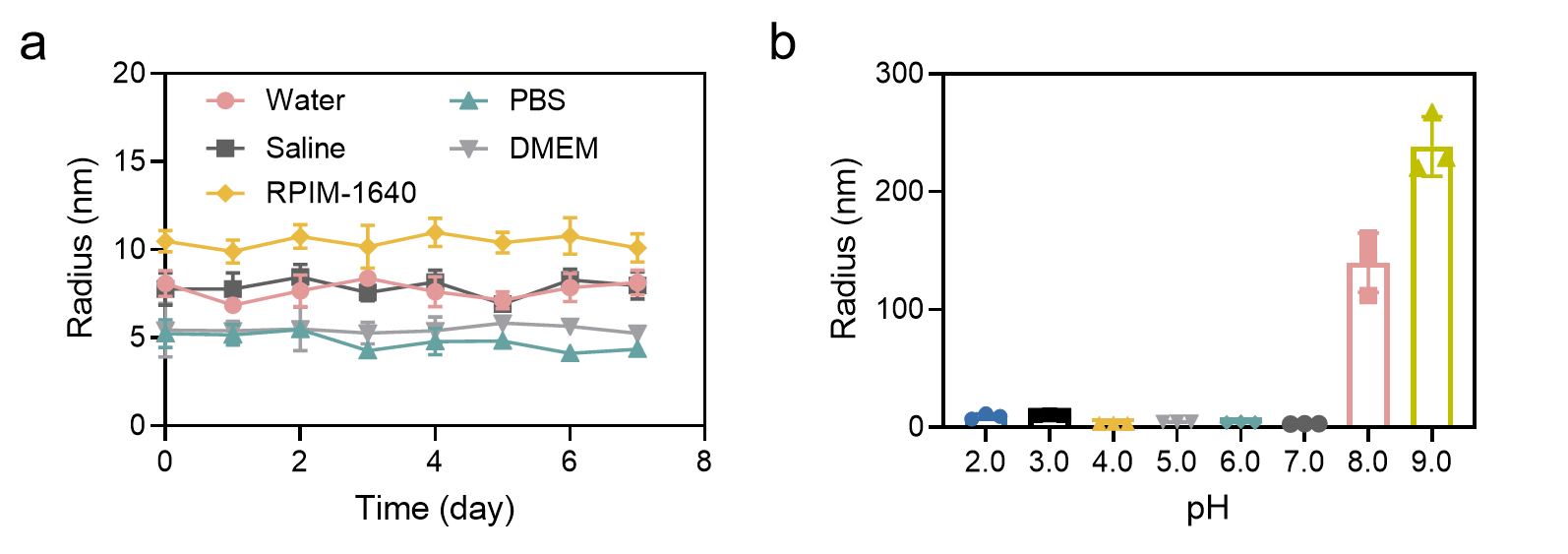


**Figure S3.** Stability of CuPaeNs under both weakly acidic conditions (pH 2.0-7.0) and during long-term storage (one week) (*n* = 3).


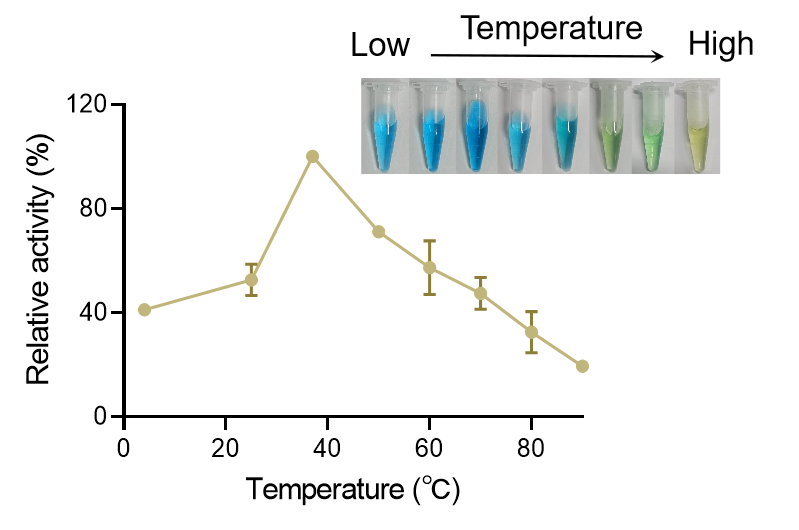


**Figure S4.** Temperature-dependent POD-like activity of CuPaeNs (*n* = 3). [TMB] = 0.832 mM, [H_2_O_2_] = 300 mM, [CuPaeNs] = 50 μg mL^-1^


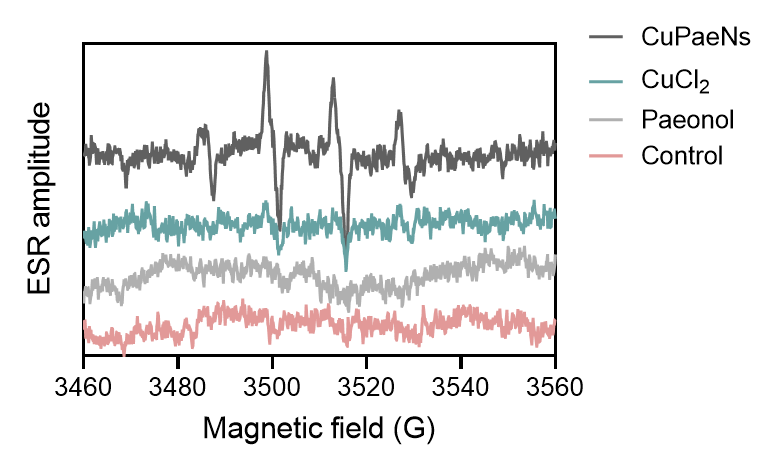


**Figure S5.** Generation of •OH by CuPaeNs in presence of 1.0 mM H_2_O_2_, determined by ESR.


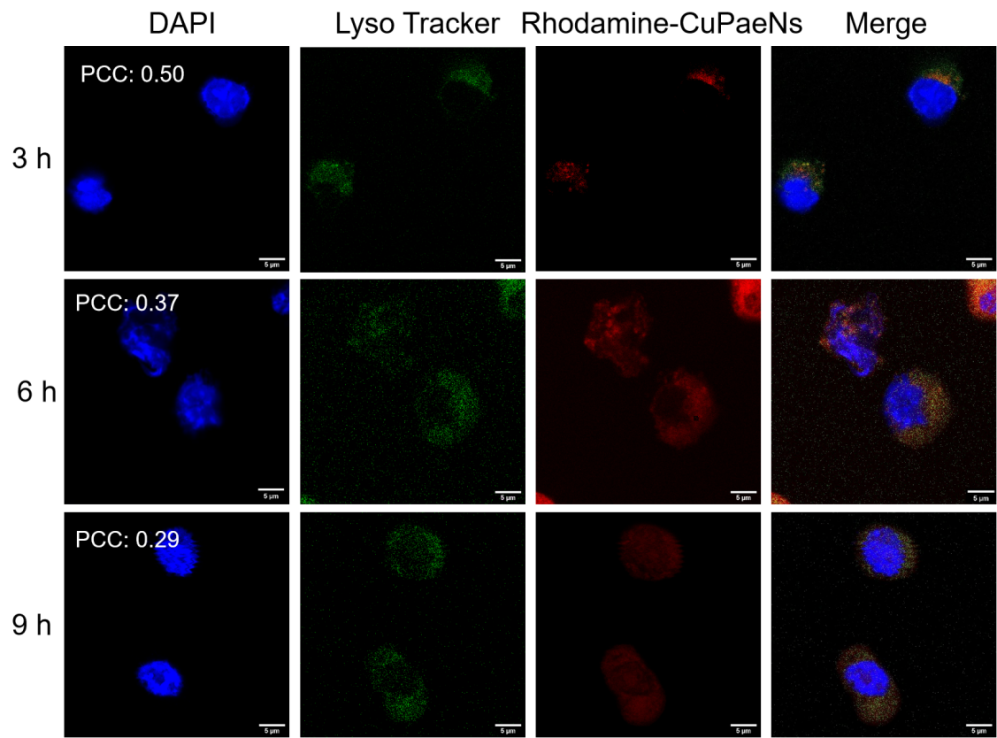


**Figure S6**. Representative CLSM images and the corresponding PCC values of Rhodamin-labeled CuPaeNs with lysosomes after incubation with B16 cells for different hours. Scale bar = 5 μm.


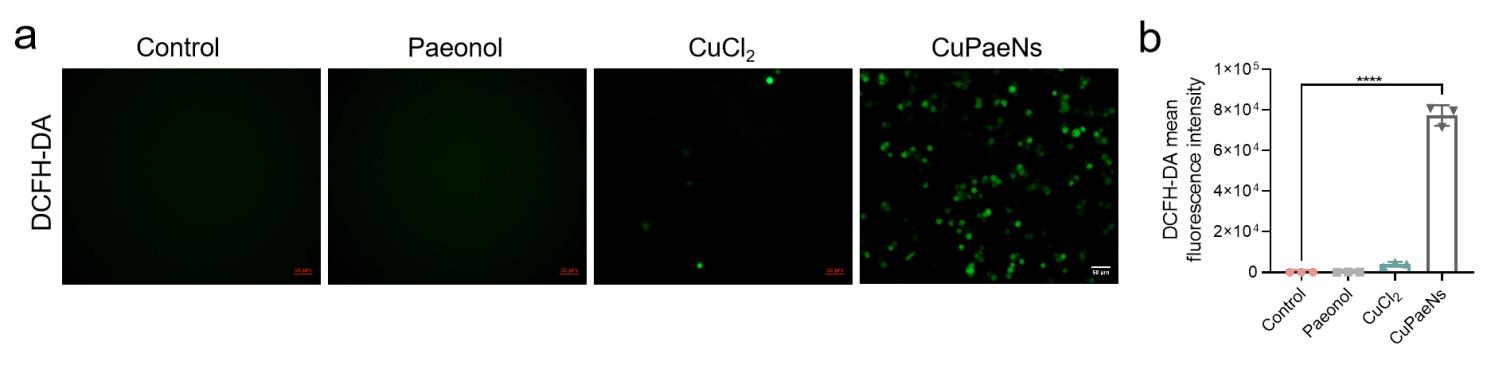


**Figure S7.** (a) Representative fluorescence images (scale bar = 50 μm) and (b) corresponding quantification (*n* = 3) of ROS in B16 cells after different treatments. Statistically significant differences are indicated with their respective P-values (**^****^***P* < 0.0001).


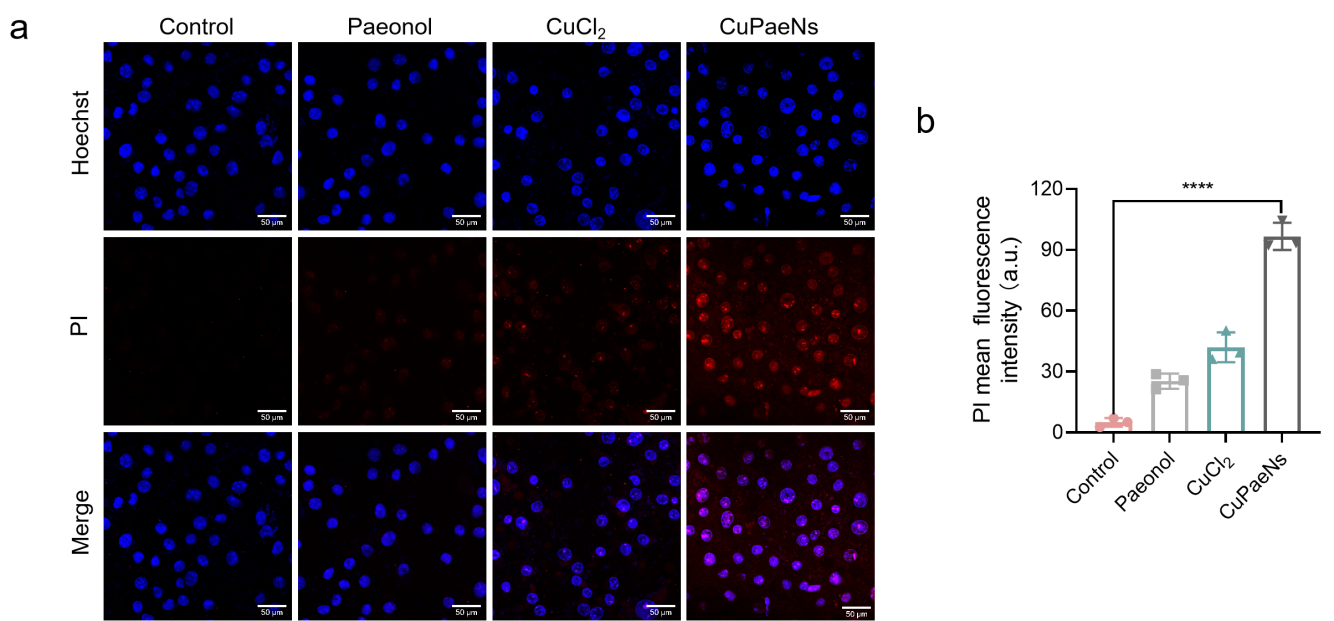


**Figure S8.** (a) CLSM images (Hoechst/PI staining) (scale bar = 50 μm) and (b) corresponding quantification (*n* = 3) of B16 cells following various treatments. Statistically significant differences are indicated with their respective P-values (**^****^***P* < 0.0001).


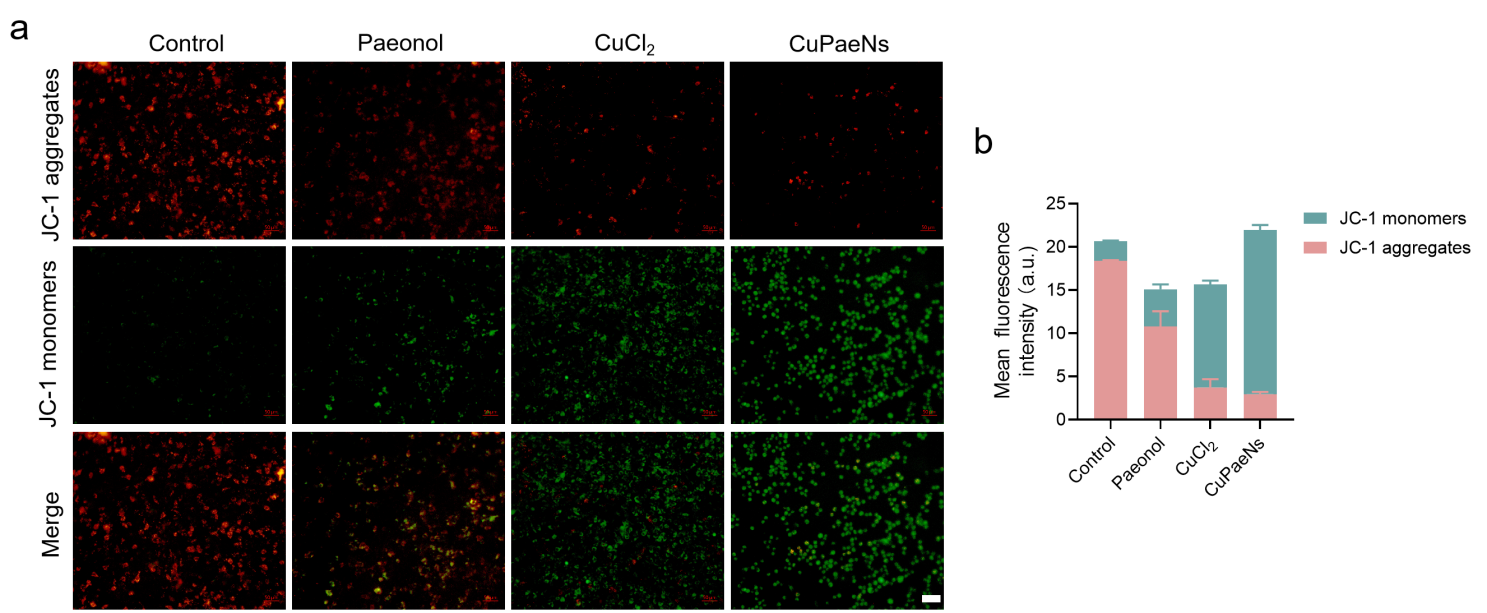


**Figure S9.** (a) JC-1 staining assay (scale bar = 50 μm) and (b) corresponding quantification (*n* = 3) of B16 cells following various treatments.


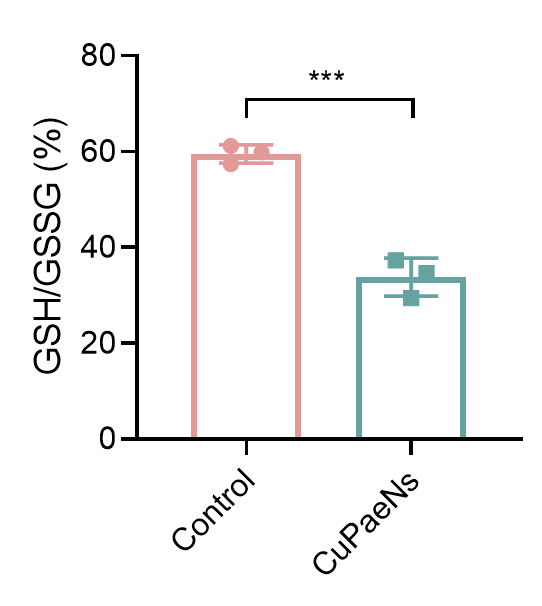


**Figure S10.** Effect of CuPaeNs on GSH/GSSG ration in B16 cells (*n* = 3). [CuPaeNs] = 250 μg/mL. Statistically significant differences are indicated with their respective P-values (**^***^***P* < 0.001).


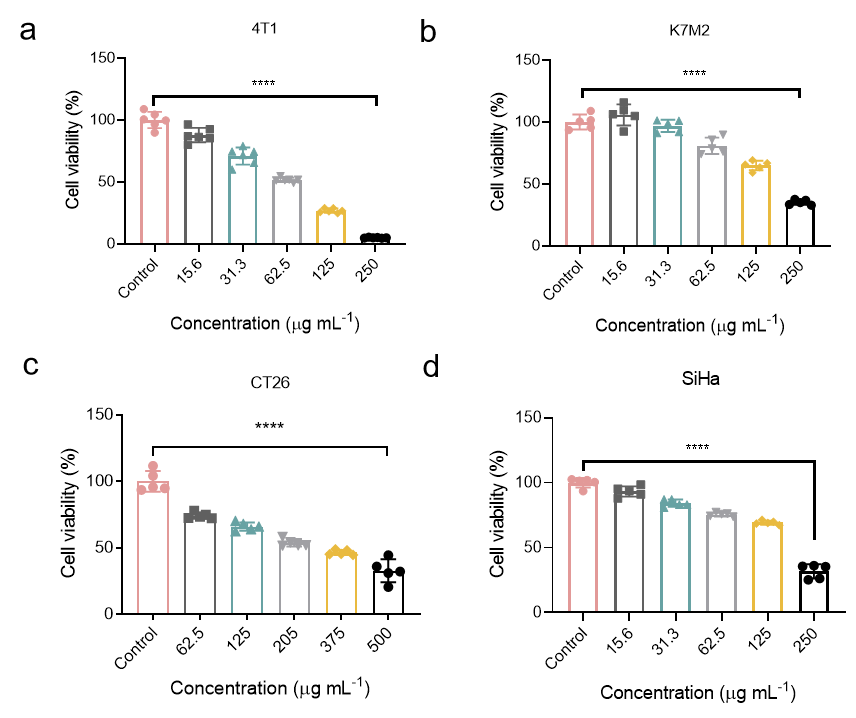


**Figure S11.** Viability of (a) 4T1, (b) K7M2, (c) CT26, (d) SiHa cells treated with various concentrations of CuPaeNs (*n* = 5). Statistically significant differences are indicated with their respective P-values (**^****^***P* < 0.0001).


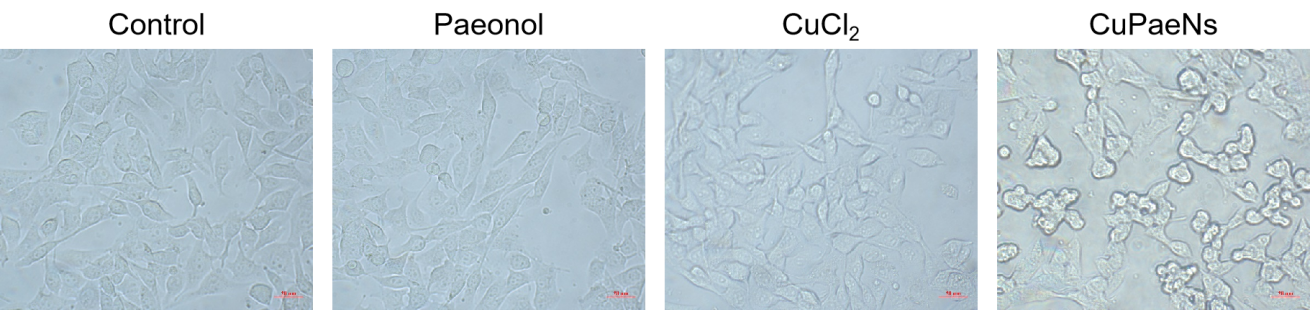


**Figure S12.** Cytomorphological changes of B16 cells after different treatments (scale bar = 20 μm).


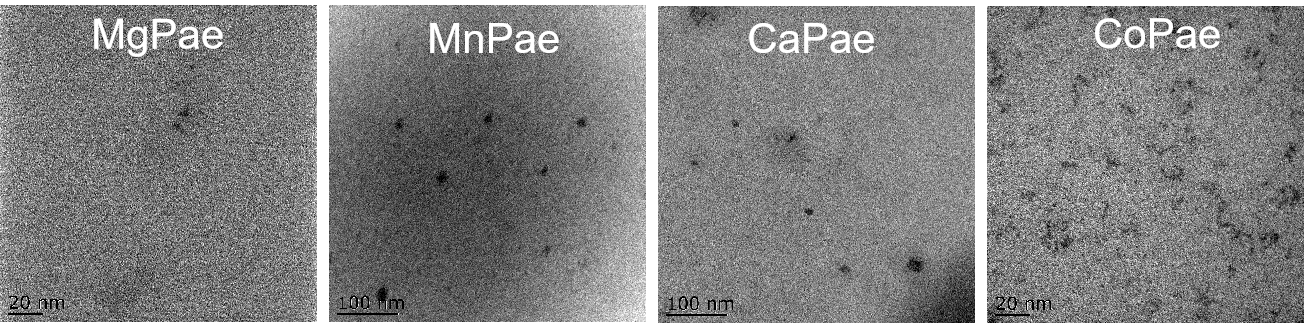


**Figure S13.** TEM images of MgPae, MnPae, CaPae, and CoPae.


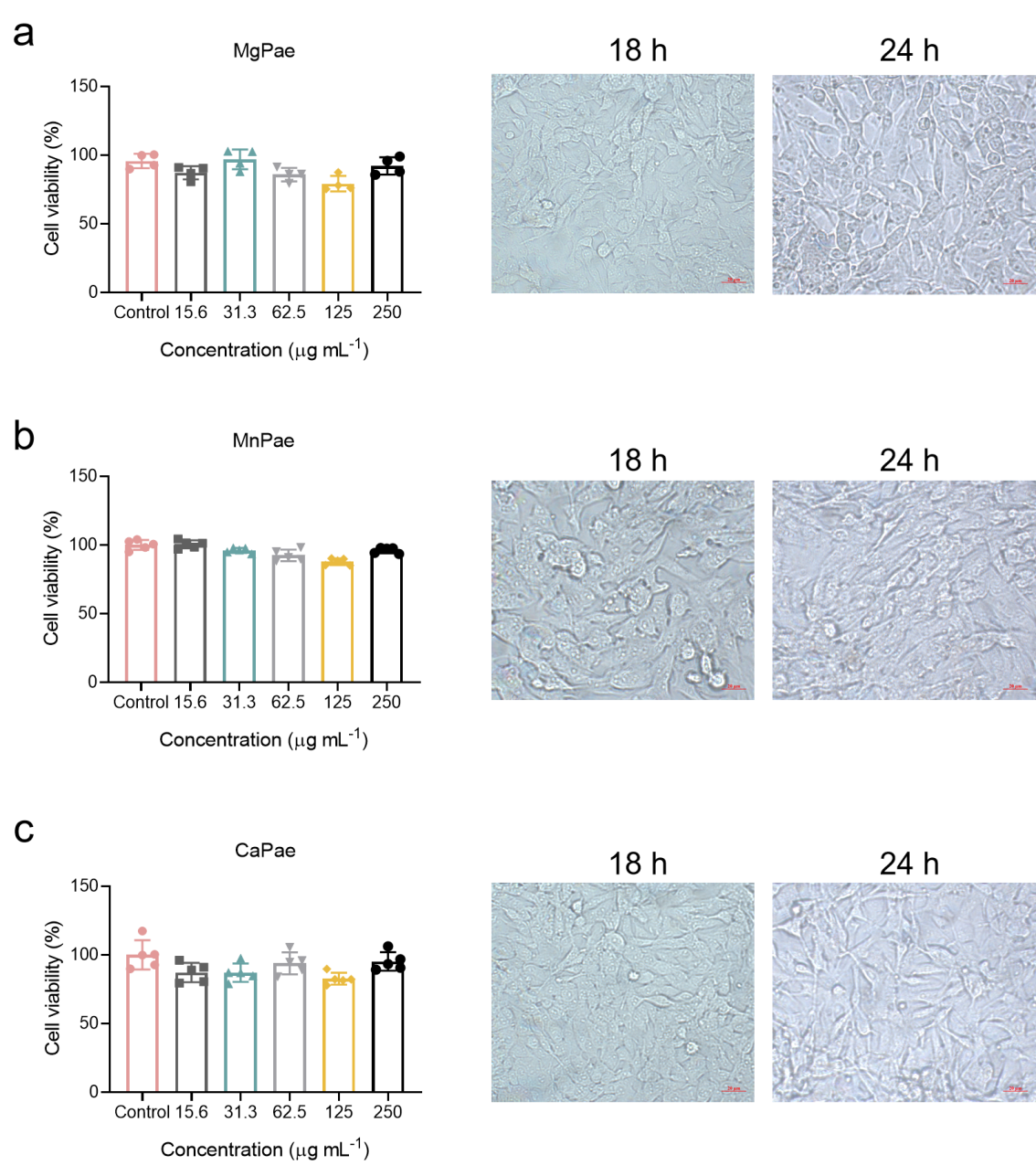


**Figure S14.** Cell viabilities (*n* = 5) and morphological changes (scale bar = 50 μm) of B16 cells after treatment with MnPae (a), MgPae (b), and CaPae (c), respectively.


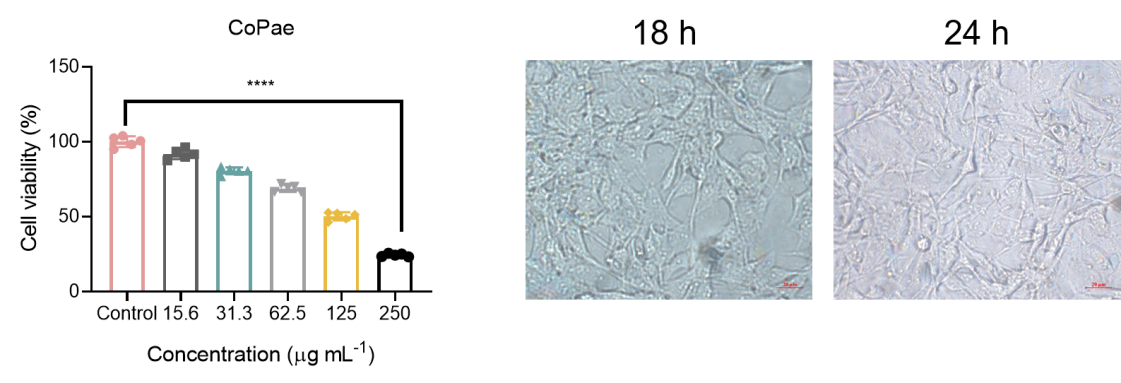


**Figure S15.** Cell viabilities (*n* = 5) and morphological changes (scale bar = 50 μm) of B16 cells after treatment with CoPae. Statistically significant differences are indicated with their respective P-values (**^****^***P* < 0.0001).


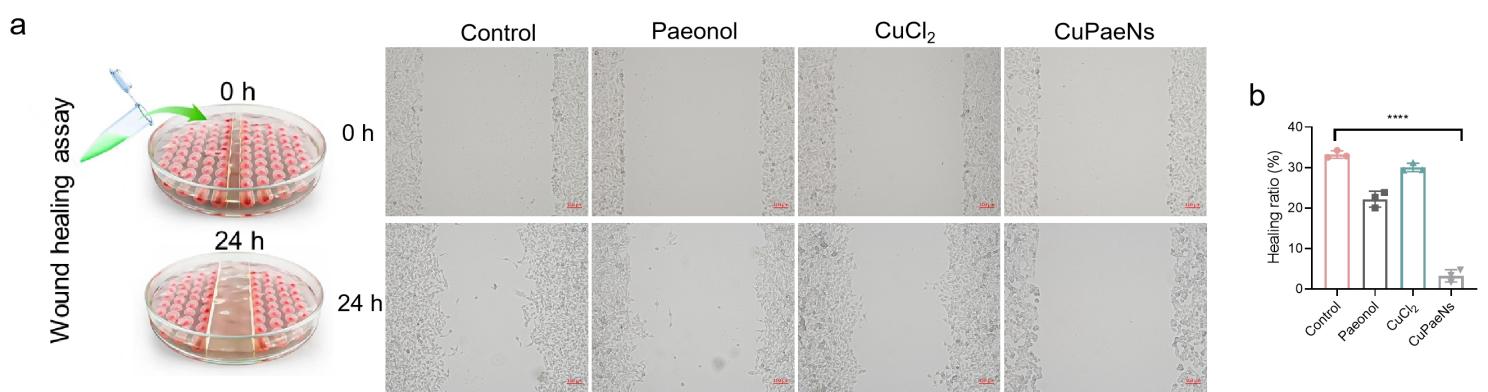


**Figure S16.** (a) Images from the wound scratch migration assay (scale bar = 100 μm) and (b) corresponding quantification (*n* = 3) of B16 cells in different groups. Statistically significant differences are indicated with their respective P-values (**^****^***P* < 0.0001).


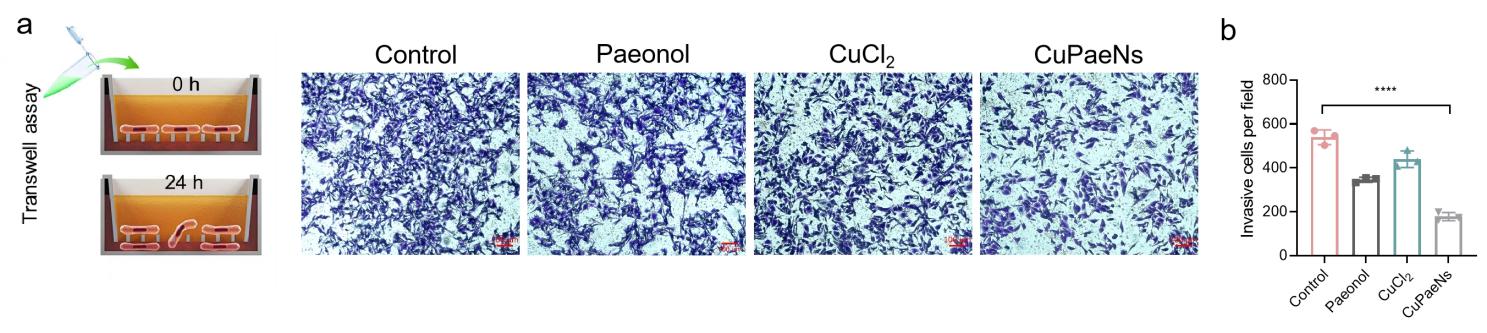


**Figure S17.** (a) Images from the transwell invasion assay (scale bar = 100 μm) and (b) corresponding quantification (*n* = 3) of B16 cells in different groups. Statistically significant differences are indicated with their respective P-values (**^****^***P* < 0.0001).


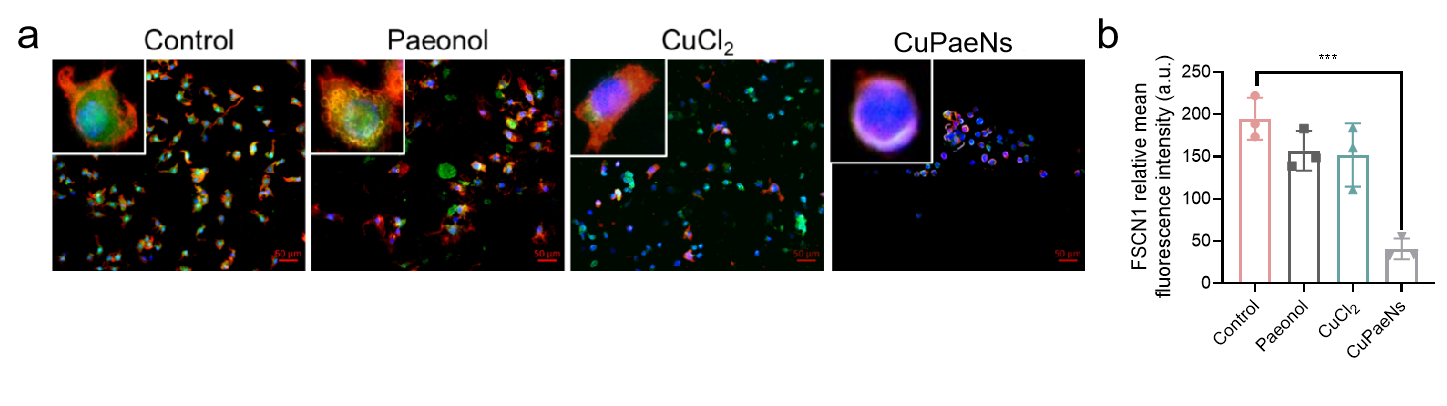


**Figure S18.** (a) Immunofluorescence staining (DAPI: blue, FSCN1: green, and F-actin: red, scale bar = 50 μm) and (b) corresponding quantification (*n* = 3) of B16 cells in different groups. Statistically significant differences are indicated with their respective P-values (**^***^***P* < 0.001,).


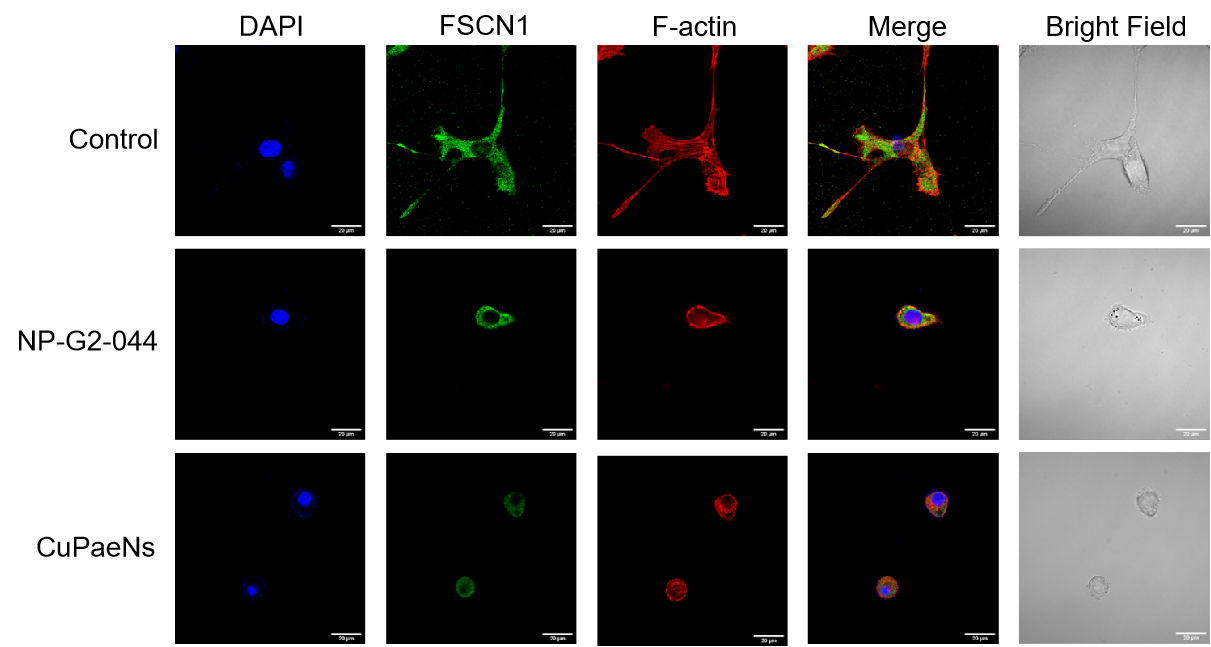


**Figure S19.** Representative CLSM images of 4T1 cells after treatments of NP-G2-044 and CuPaeNs (scale bar = 20 μm).


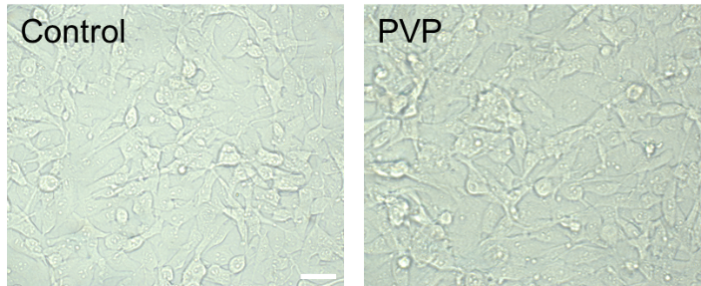


**Figure S20.** Morphological changes of B16 cells after treatment with PVP (scale bar = 20 μm).


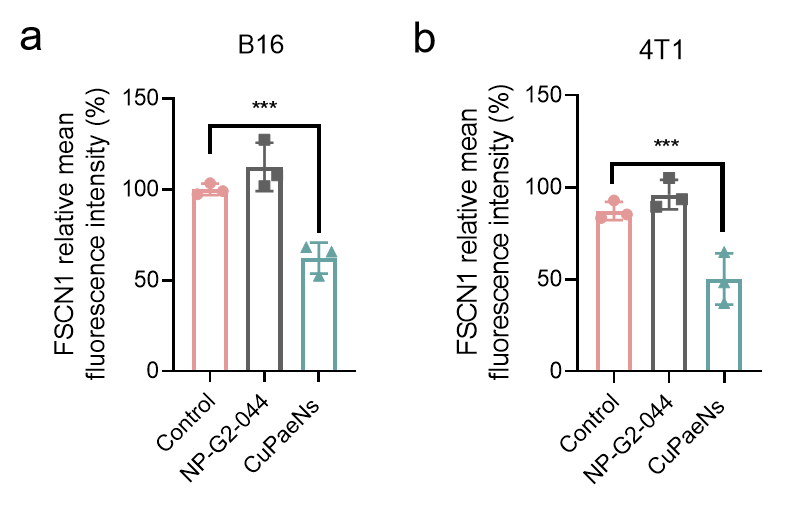


**Figure S21.** Corresponding FSCN1 relative mean fluorescence intensity of CLSM images of (a) B16 and (b) 4T1 cells treated with PBS (Control), NP-G2-044 and CuPaeNs (*n* = 3). Statistically significant differences are indicated with their respective P-values (**^***^***P* < 0.001).


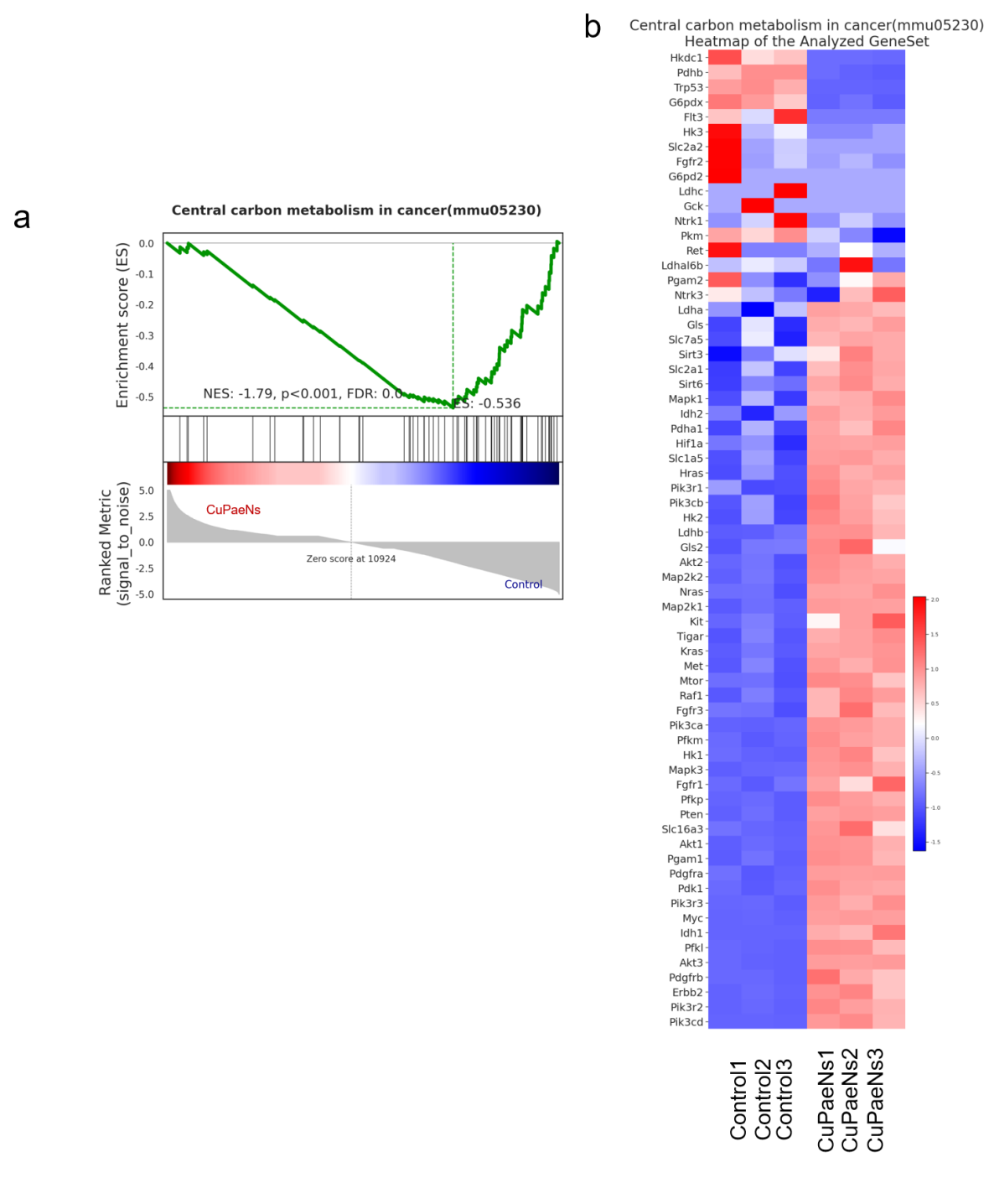


**Figure S22.** (a) GSEA analysis and (b) heatmap of central carbon metablism signaling pathway in B16 Cells. NES and FDR were indicated.


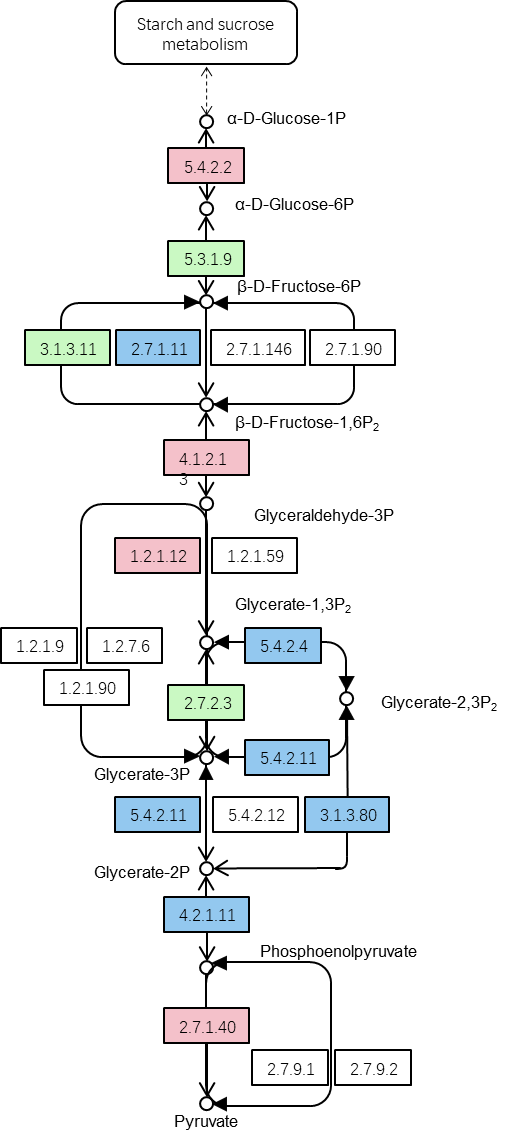


**Figure S23.** Schematic of CuPaeNs inhibiting glycolysis.


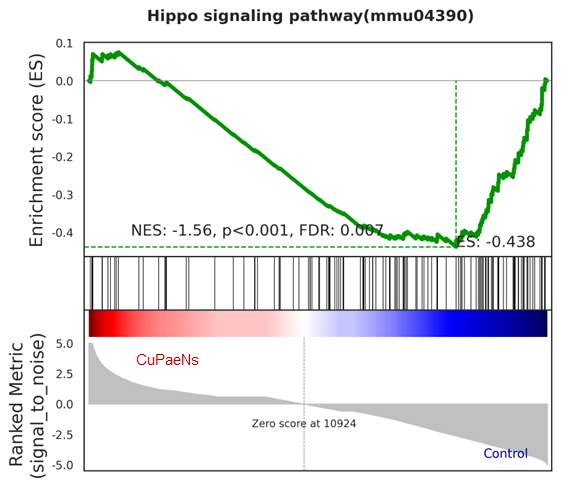


**Figure S24.** (a) GSEA analysis of Hippo signaling pathway in B16 Cells. NES and FDR were indicated.


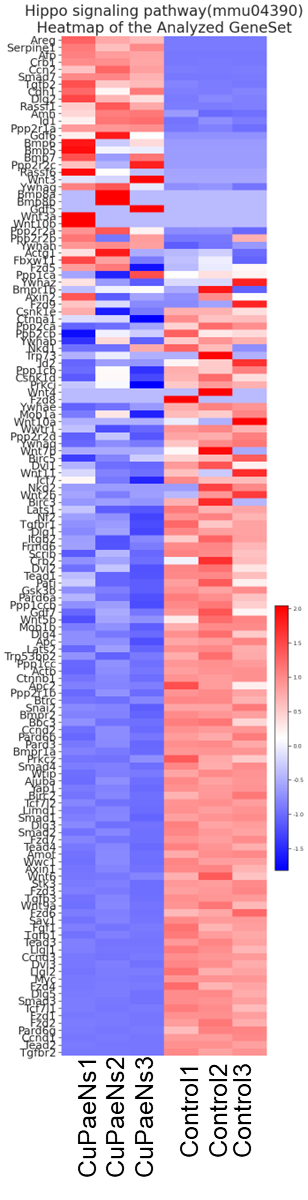


**Figure S25.** Heatmap of Hippo signaling pathway in B16 Cells

.
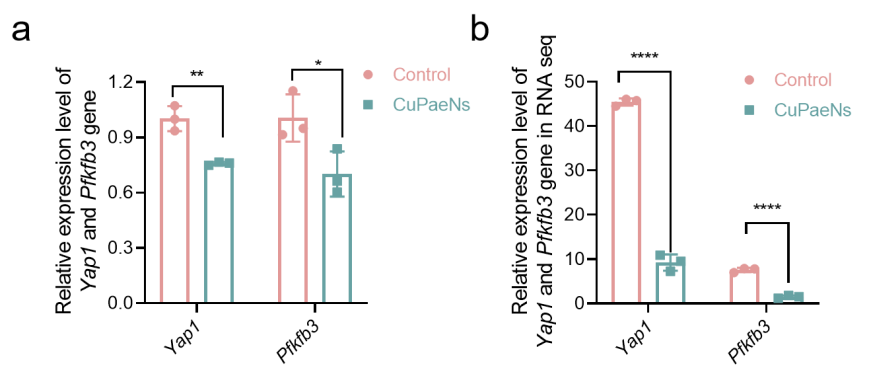


**Figure S26.** (a) Relative mRNA expression levels of *Yap1* and *Pfkfb3* determined by qPCR (*n* = 3).

(b) Corresponding RNA-seq expression profiles (*n* = 3). Statistically significant differences are indicated with their respective P-values (**^*^***P* < 0.05, **^**^***P* < 0.01, **^****^***P* < 0.0001).


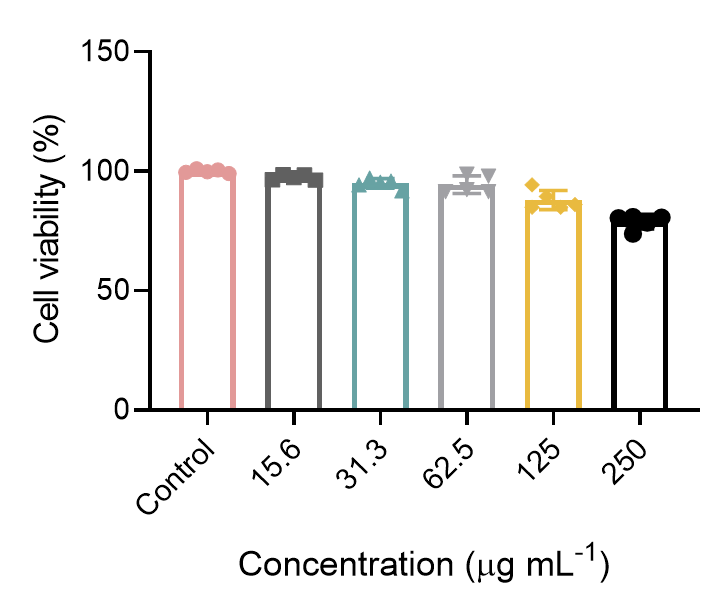


**Figure S27**. Viability of L02 cells following treatment with varying concentrations of CuPaeNs (*n* = 5).


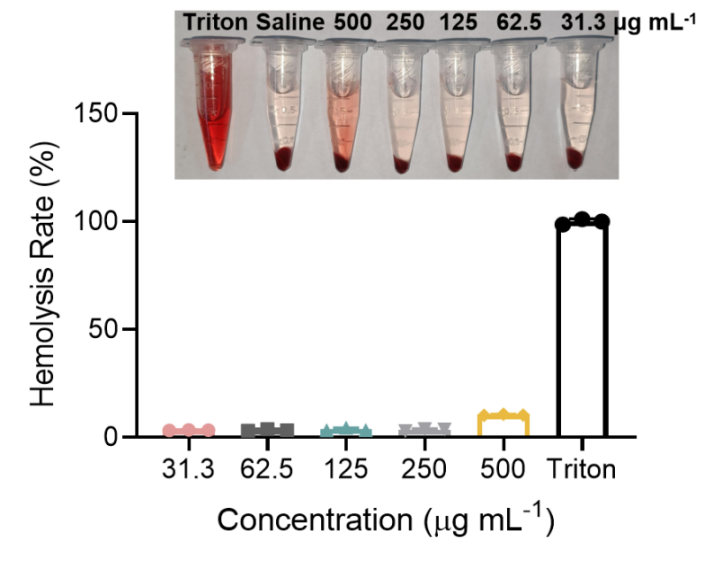


**Figure S28.** Hemolysis evaluation of CuPaeNs (*n* = 3).


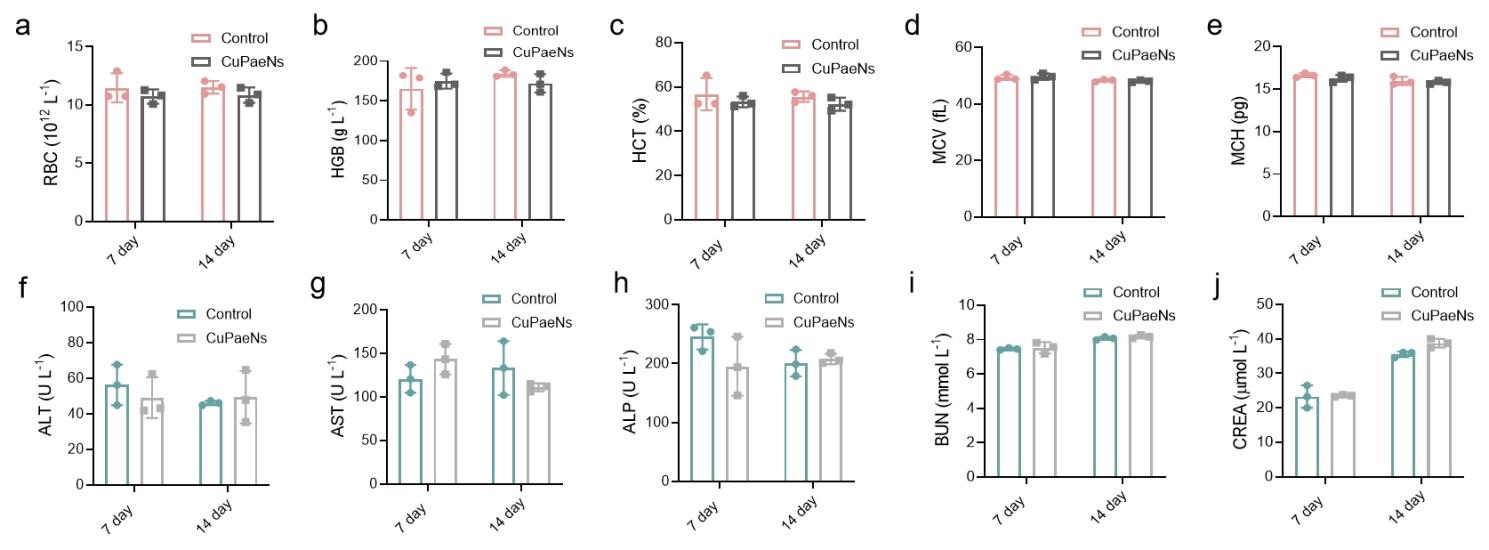


**Figure S29.** (a-e) Blood routine data obtained from i.v. treatment with CuPaeNs on day 7 and day 14, including RBC, HGB, HCT, MCV, and MCH (*n* = 3). (f-j) Serum biochemistry data obtained from i.v. treatment with CuPaeNs on day 7 and day 14, including liver function (ALP, ALT, and AST) and kidney function (CREA and BUN) (*n* = 3).


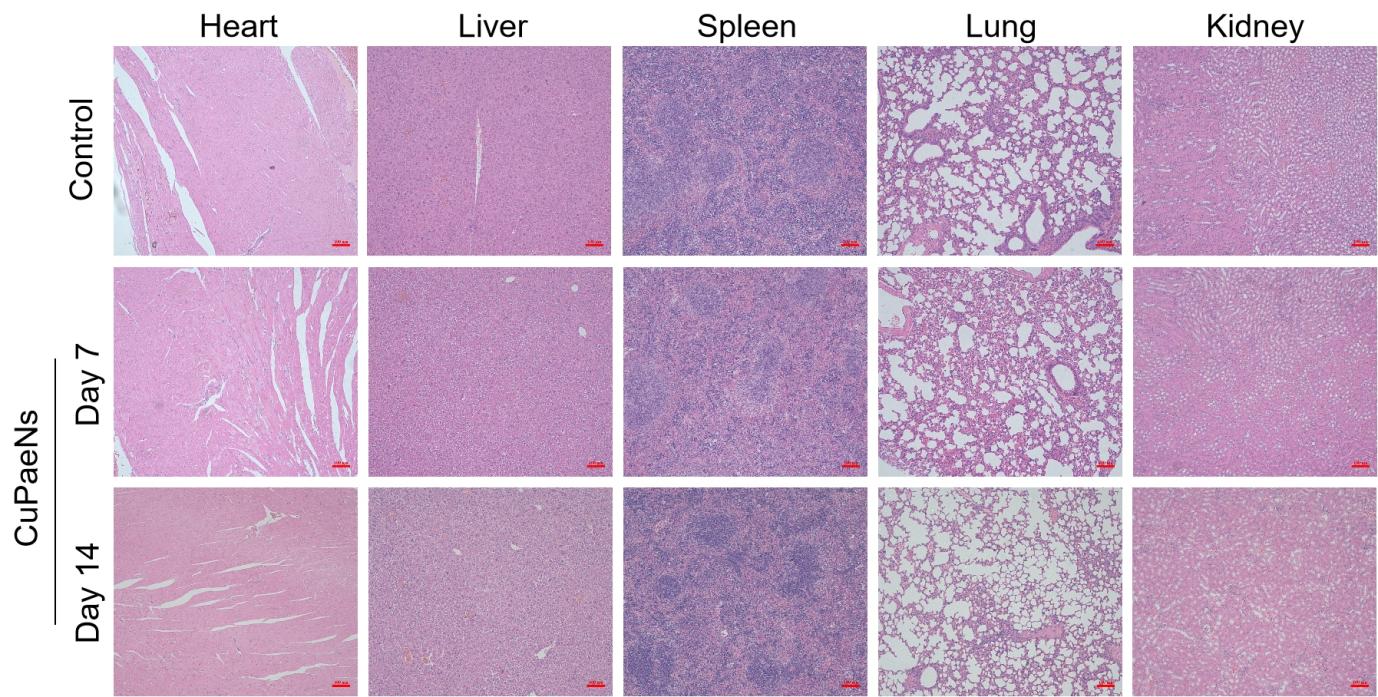


**Figure S30.** H&E staining of the major organs (heart, liver, spleen, lung, and kidney) from healthy mice following i.v. injection of CuPaeNs on day 7 and 14 (scale bar = 100 µm).


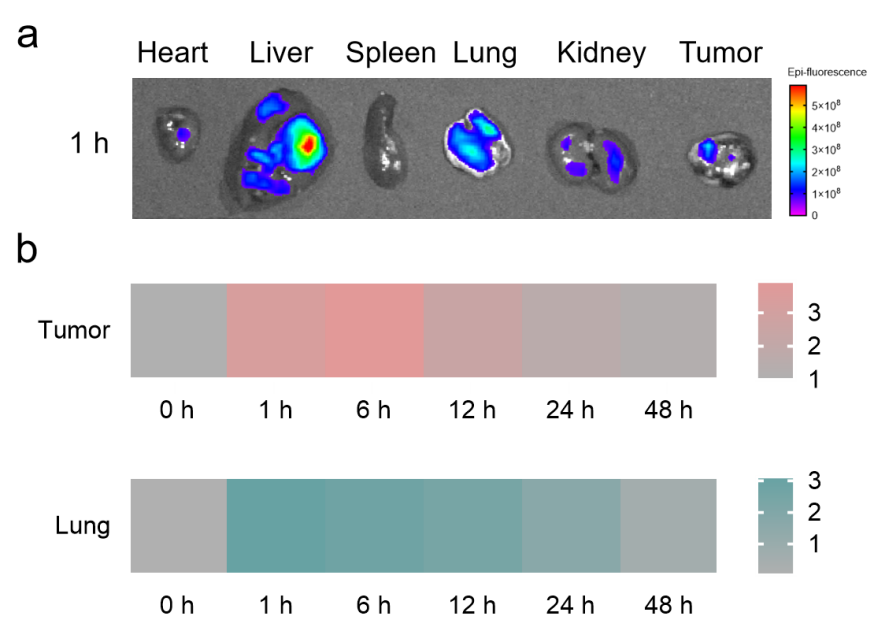


**Figure S31.** *In vivo* bdistribution of CuPaeNs. (a) Representative *in vivo* fluorescence images of mice at 1 h post intravenous injection. (b) Biodistribution quantified by ICP-MS, measuring copper levels in lung and tumor tissues at designated time points, normalized to the copper content at 0 h (*n* = 3).

**Figure S32.** Observation of body weight changes in subcutaneous tumor model over 16 day of treatment (*n* = 5).


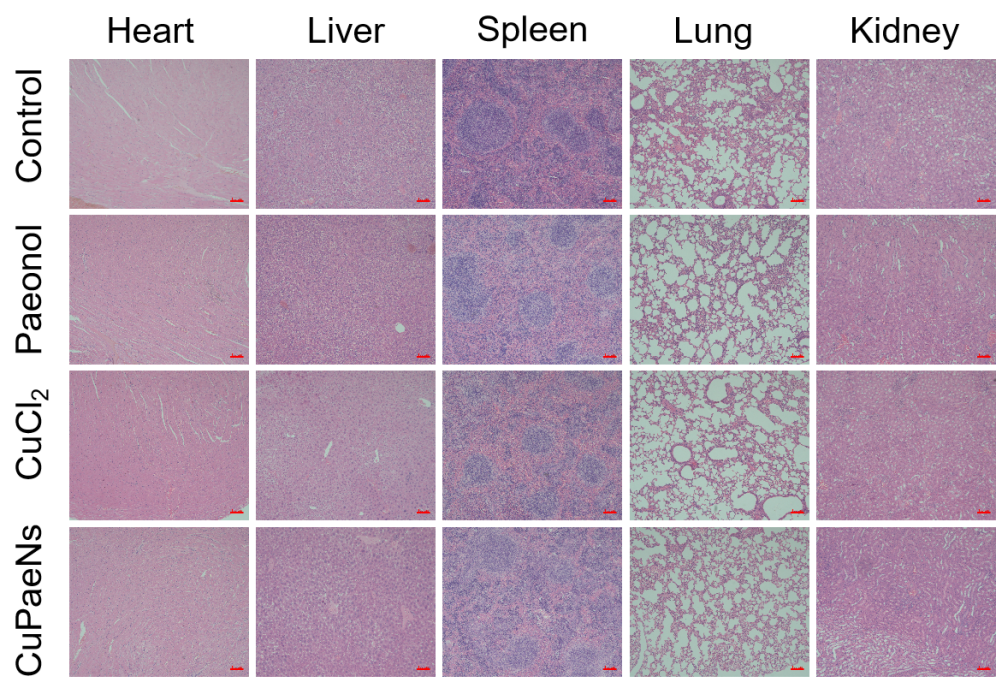


**Figure S33.** H&E staining of the major organs (heart, liver, spleen, lung, and kidney) from B16-bearing mice following different treatments (scale bar = 100 µm).


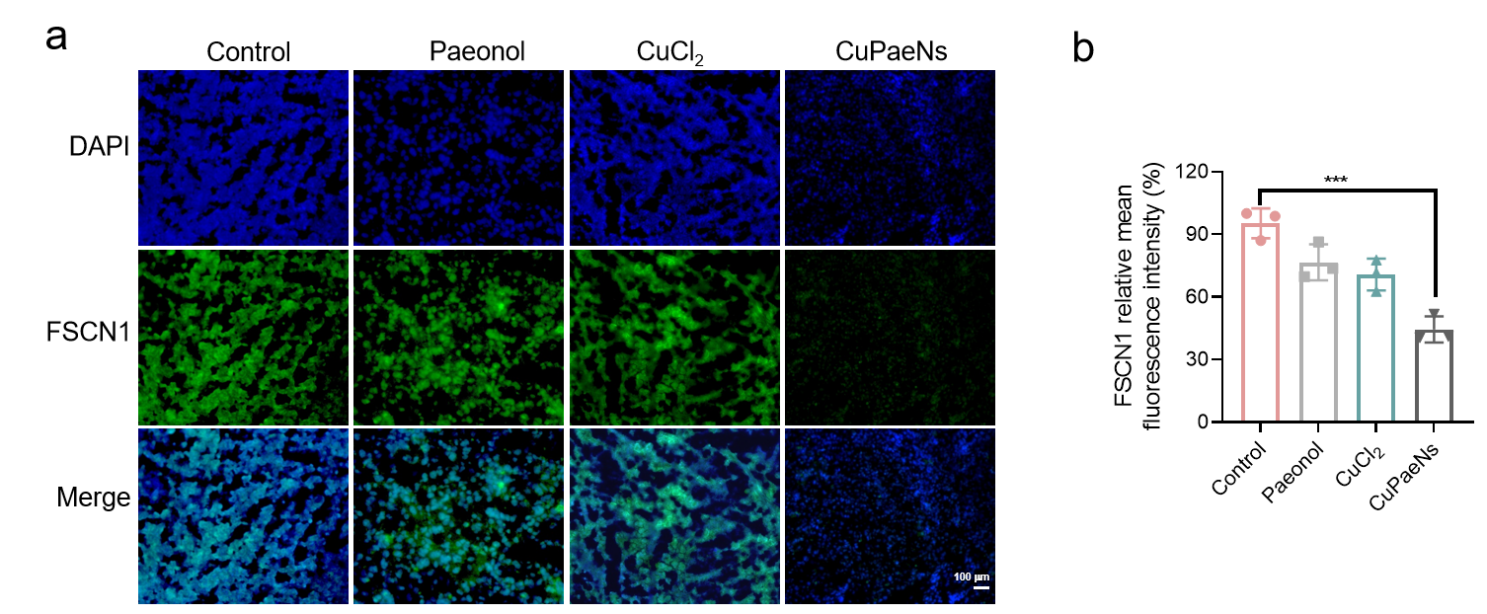


**Figure S34.** (a) Representative immunofluorescence images (scale bar = 20 μm) and (b) corresponding quantification (*n* = 3) showing the expression of FSCN1 (green) in tumor treated with PBS, paeonol, CuCl_2_, and CuPaeNs, respectively. Statistically significant differences are indicated with their respective P-values (**^***^***P* < 0.001).


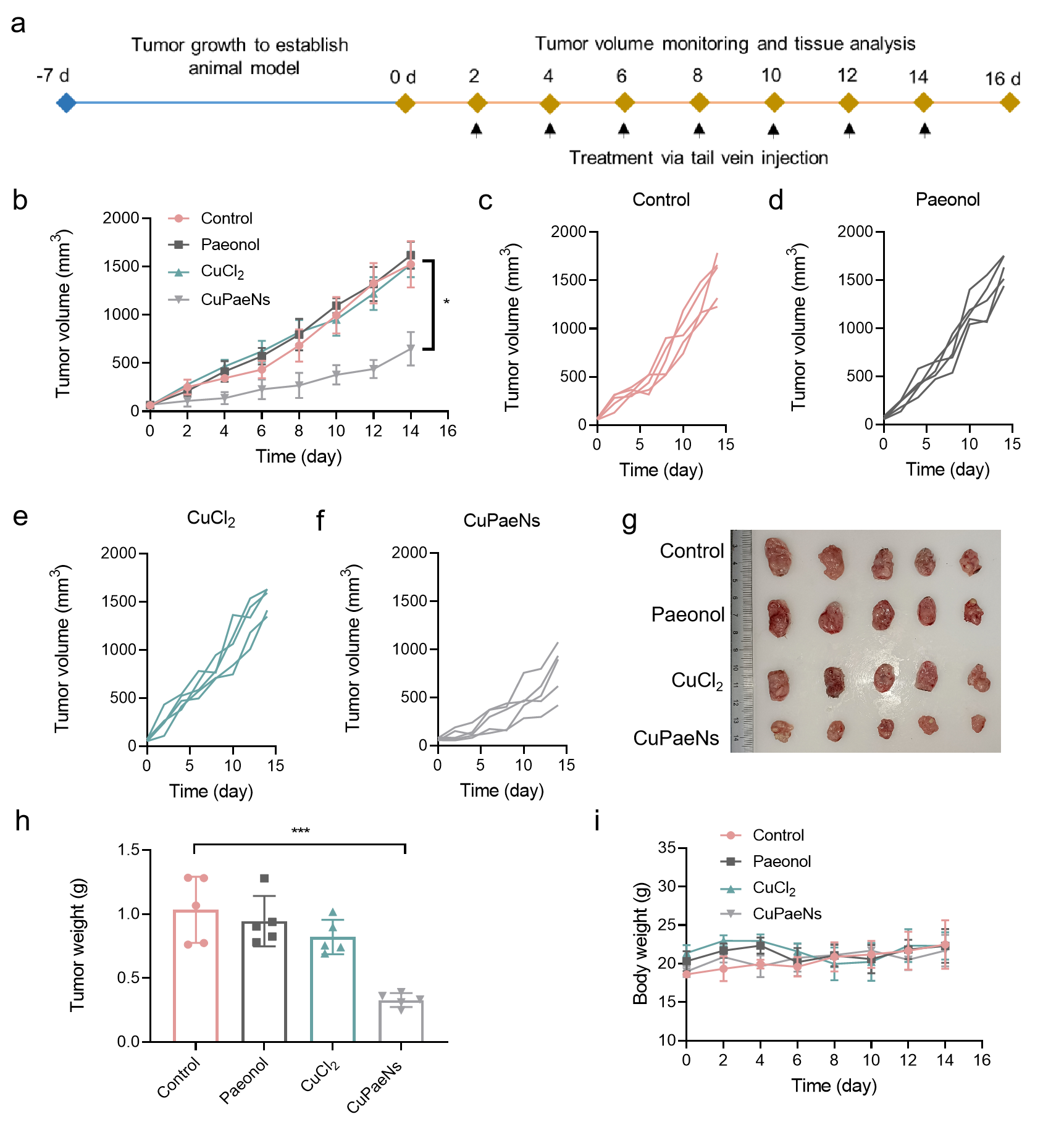


**Figure S35.** (a) Schematic illustration of tumor inoculation and treatment protocol for 4T1 xenograft tumor-bearing mice. (b-f) Observation of tumor inhibitory effect of mice in subcutaneous tumor model over 16 days of treatment (*n* = 5). (g) Representative images of harvested 4T1 tumors and (h) tumor weights after treatment with PBS, paeonol, CuCl_2_, and CuPaeNs, respectively (*n* = 5). (i) Observation of body weight changes in subcutaneous tumor model over 16 days of treatment (*n* = 5). Statistically significant differences are indicated with their respective P-values (**^*^***P* < 0.05 , **^***^***P* < 0.001).

**Table S1.** Comparison of POD-like catalytic kinetic parameters between CuPaeNs and other copper-based nanozymes using H_2_O_2_ as substrate.


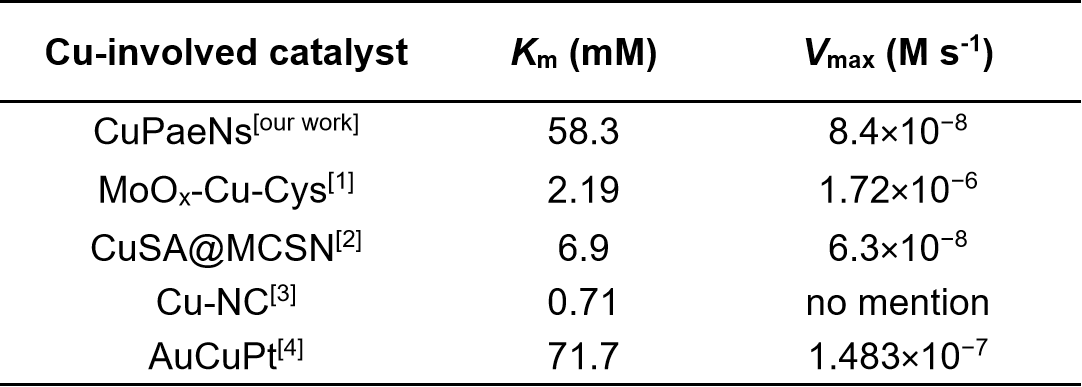


**Table S2.** Sequences of primer (5' to 3').


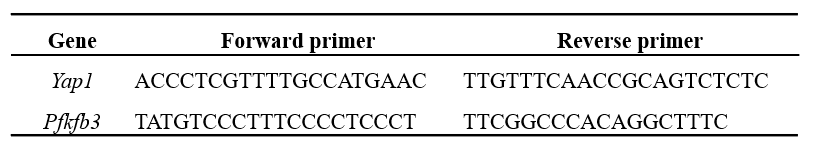


**References**

[1] Ma B, Wang S, Liu F, Zhang S, Duan J, Li Z, Kong Y, Sang Y, Liu H, Bu W, Li L, Self-assembled copper-amino acid nanoparticles for in situ glutathione "AND" H_2_O_2_ sequentially triggered chemodynamic therapy. *J. Am Chem Soc.* 2019, 141(2), 849-857.

[2] Xu S, Yuan Y, Pan Y, Zhang H, Yin S, Zhang X, Zhang Y, Dai S, Shen H, Li Y, Niu D, A highly efficient and biocompatible cupper-based single-atom nanocatalyst with unsaturated coordination structure for efficient diabetic wound healing. *Small* 2025, 21(31), e2502896.

[3] Zhu J, Li Q, Li X, Wu X, Yuan T, Yang Y, Simulated enzyme activity and efficient antibacterial activity of copper-doped single-atom nanozymes. *Langmuir*, 2022, 38(22), 6860-6870.

[4] Liu J, Dong S, Gai S, et al. Design and mechanism insight of monodispersed AuCuPt alloy nanozyme with antitumor activity. *ACS Nano* 2023, 17(20), 20402-20423.
